# A xonotlite nanofiber bioactive 3D-printed hydrogel scaffold based on osteo-/angiogenesis and osteoimmune microenvironment remodeling accelerates vascularized bone regeneration

**DOI:** 10.1186/s12951-024-02323-9

**Published:** 2024-02-12

**Authors:** Shi-Yuan Yang, Yu-Ning Zhou, Xing-Ge Yu, Ze-Yu Fu, Can-Can Zhao, Yue Hu, Kai-Li Lin, Yuan-Jin Xu

**Affiliations:** 1 Department of Oral Surgery, Shanghai Ninth People’s Hospital, Shanghai Jiao Tong University School of Medicine, 639 Zhizaoju Road, Shanghai, 200011 China; 2 College of Stomatology, National Center for Stomatology; National Clinical Research Center for Oral Diseases; Shanghai Key Laboratory of Stomatology; Shanghai Research Institute of Stomatology, Shanghai Jiao Tong University, Shanghai, China; 3 Department of Oral and Cranio-Maxillofacial Surgery, Shanghai Ninth People’s Hospital, Shanghai Jiao Tong University School of Medicine, Shanghai, China

**Keywords:** Xonotlite nanofiber, 3D printed, Bone regeneration, Macrophages, Osteoimmune microenvironment remodeling

## Abstract

**Background:**

Coordination between osteo-/angiogenesis and the osteoimmune microenvironment is essential for effective bone repair with biomaterials. As a highly personalized and precise biomaterial suitable for repairing complex bone defects in clinical practice, it is essential to endow 3D-printed scaffold the above key capabilities.

**Results:**

Herein, by introducing xonotlite nanofiber (Ca_6_(Si_6_O_17_) (OH)_2_, CS) into the 3D-printed silk fibroin/gelatin basal scaffold, a novel bone repair system named SGC was fabricated. It was noted that the incorporation of CS could greatly enhance the chemical and mechanical properties of the scaffold to match the needs of bone regeneration. Besides, benefiting from the addition of CS, SGC scaffolds could accelerate osteo-/angiogenic differentiation of bone mesenchymal stem cells (BMSCs) and meanwhile reprogram macrophages to establish a favorable osteoimmune microenvironment. In vivo experiments further demonstrated that SGC scaffolds could efficiently stimulate bone repair and create a regeneration-friendly osteoimmune microenvironment. Mechanistically, we discovered that SGC scaffolds may achieve immune reprogramming in macrophages through a decrease in the expression of Smad6 and Smad7, both of which participate in the transforming growth factor-β (TGF-β) signaling pathway.

**Conclusion:**

Overall, this study demonstrated the clinical potential of the SGC scaffold due to its favorable pro-osteo-/angiogenic and osteoimmunomodulatory properties. In addition, it is a promising strategy to develop novel bone repair biomaterials by taking osteoinduction and osteoimmune microenvironment remodeling functions into account.

**Graphical Abstract:**

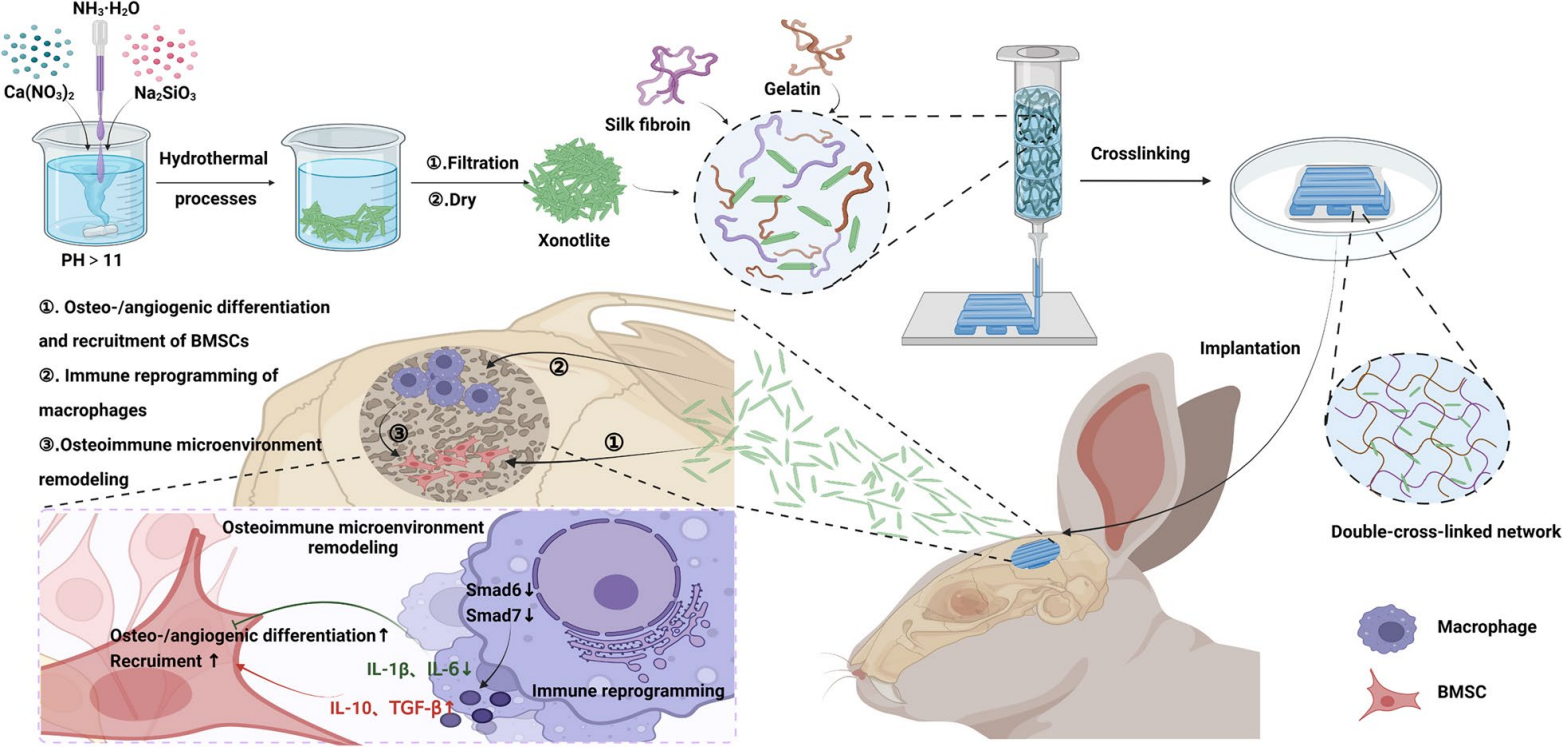

**Supplementary Information:**

The online version contains supplementary material available at 10.1186/s12951-024-02323-9.

## Introduction

Clinical intervention is needed to repair large bone defects resulting from high-energy trauma, bone tumor removal, and osteomyelitis debridement [1–3]. Despite traditional treatments such as autobone transplantation (the golden standard) and heterogeneous transplantation are widely applied in clinical at present, it could cause plenty of risks during and after the surgery [4]. Therefore, there is an urgent need for the development of biomaterials for better clinical treatment of bone defect.

In recent years, 3D printing technology has been widely adopted to produce printed biomaterials for bone repair due to its advantages of controllability, precision and personalization [5, 6]. In which, 3D-printed hydrogels have attracted much attention in the field of bone regeneration, considering their 3D network similar to the extracellular matrix (ECM) [7]. Like most biomaterial implants, printed hydrogels trigger a host immune response that either benefits or hinders bone regeneration process [8–11]. Therefore, besides their pro-osteo-/angiogenic effects on BMSCs, novel printed hydrogels should also be evaluated for their "osteoimmunomodulation" ability, that is, whether they can regulate the local immune response to form an optimal osteoimmune microenvironment conducive to bone regeneration. In addition, the number of endogenous BMSCs present at the injury site is frequently inadequate, thereby reducing the effectiveness of the osteo-/angiogenic capabilities of the printed hydrogels. Thus, optimal printed hydrogels preferably include the ability to recruit endogenous BMSCs [12, 13].

Macrophages, a key player in the immunological response caused by foreign implants, are classified into two classical subtypes of M1 and M2 phenotypes [14]. The phenotype reprogramming of macrophages not only regulates local inflammatory response, but also remodels the osteoimmune microenvironment to further influence the osteo-/angiogenic differentiation of BMSCs [15]. As is known to all, the pro-inflammatory M1 phenotype could activate inflammation, promote fibrotic encapsulation, and construct a harmful osteoimmune microenvironment, ultimately leading to implant failure and poor bone regeneration, whereas the M2 phenotype has the opposite effects [16–18]. Therefore, reprogramming macrophages to inhibit M1 polarization and promote M2 polarization as well as remodeling the osteoimmune microenvironment might be of great importance for printed hydrogel employed in bone regeneration.

Currently, the hydrogels commonly used for 3D printing, such as silk fibroin (SF) and gelatin, have been reported to have limited capabilities for bone regeneration including osteo-/angiogenesis and immune regulation, while their combination has attractive mechanical strength and controllable degradation rate [12, 19–21]. CS, as a fibrous or needle-like inorganic material, could induce osteo-/angiogenic differentiation of BMSCs by continuously releasing Ca^2+^ and Si^4+^ bioactive ions, which were contributed to promoting bone regeneration and vessel formation [1, 22–24]. In addition, research have shown that CS also could reprogram macrophages toward M2 phenotype [25]. As a result of the findings presented above, we hypothesized that incorporating CS into the base ink (SF and gelatin) would produce 3D-printed scaffolds that combine the advantageous properties of these materials, which may be favorable for treating bone defect.

Inspired by above biological knowledge, a 3D-printed composite hydrogel scaffold incorporated with CS was fabricated to achieve vascularized bone regeneration in situ. First, we synthesized CS by hydrothermal method and then added it to the base ink (SF and gelatin). Thereafter, based on 3D printing technology, the composite ink was extruded and then cross-linked with genipin and ethanol to obtain the 3D-printed composite porous hydrogel scaffold. Benefiting from the addition of CS, the SGC scaffold as a novel bone repair system has the following advantages: (1) the SGC scaffold possessed satisfactory chemical and mechanical properties for bone repair purposes; (2) the SGC scaffold could stimulate the osteo-/angiogenesis of BMSCs and reprogram macrophages towards M2 phenotype; (3) the SGC scaffold could strength the recruitment and osteo-/angiogenesis of BMSCs via remodeling osteoimmune microenvironment. To sum up, the above work not only proposed a novel bone repair scaffold called SGC, which could effectively achieve bone regeneration in situ, but also highlighted the need to consider the coordination of osteo-/angiogenesis and osteoimmune microenvironment during the design of biomaterials.

## Results and discussion

### Characterization of CS nanowires

From Additional file 1: Fig. S1A, the X-ray diffraction (XRD) spectra showed that the obtained powders had the characteristic peaks of xonotlite (JCPDS card: No. 23-0125). The transmission electron microscope (TEM) images (Additional file 1: Fig. S1B) further confirmed that the synthesized powders had smooth surface and the shape of ultra-long wire as reported before [1, 26].

### Rheological evaluation

**Fig. 1:**
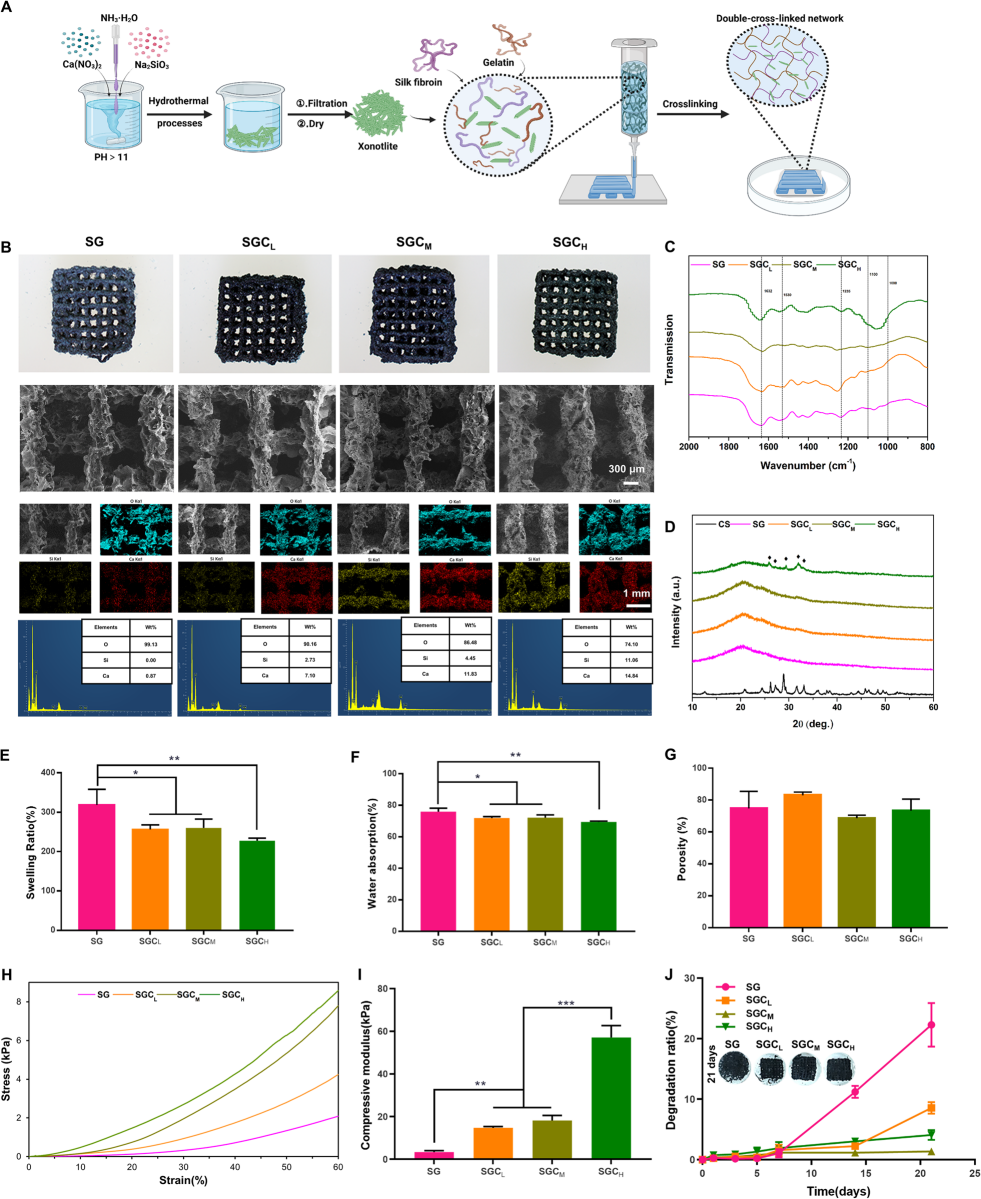
Characteristic of the 3D-printed scaffolds. **A** Schematic illustration of the manufacturing process of the xonotlite nanofiber bioactive 3D-printed hydrogel scaffold. **B** Macroscopic photographs, SEM images, element mapping and EDS of four scaffolds. **C** FTIR spectra, **D** XRD (black diamond marks the specific peaks of CS),** E** swelling ratio, **F** water absorption,** G** porosity,** H** stress–strain curve,** I** compressive modulus and** J** degradation ratio of SG, SGC_L_, SGC_M_ and SGC_H_ samples. ^*^P < 0.05, ^**^P < 0.01 or.^***^P < 0.001

To determine the optimal amount of CS addition, three SGC scaffolds with varying concentration gradients were prepared based on SF/gelatin (SG) scaffold for subsequent experiments, referred to as SGC_L_ (*w/w/w*, CS:SF:gelatin = 6.25:100:100) scaffold, SGC_M_ (*w/w/w*, CS:SF:gelatin = 12.5:100:100) scaffold and SGC_H_ (*w/w/w*, CS:SF:gelatin = 25:100:100) scaffold. Prior to printing, we examined the printability and rheological properties of the four inks (Additional file 1: Fig. S1C). The viscosity of these ink all showed shear thinning behavior at 25℃, which afforded their extrudable property and good printability as suitable 3D-printing ink [27]. The obtained ink was utilized to further prepare the xonotlite nanofiber bioactive 3D-printed hydrogel scaffold, as illustrated in Fig. 1A.

### Characterization of the 3D-printed scaffolds

#### Microscopic morphology

Macroscopic photographs and scanning electron microscopy (SEM) micrographs revealed that all four scaffolds exhibited a rough surface morphology with an average pore size of approximately 600 μm. This pore size facilitates bone ingrowth and vascularization [28–31].

The element mapping showed that Ca and Si (representing CS) were uniformly distributed in the SGC scaffolds, indicating the successful incorporation of CS. Additionally, quantitative analysis of energy dispersive spectrometer (EDS) spectra showed that the peak intensities of the Ca and Si components were directly proportional to the quantity of CS present in the scaffolds, reinforcing the success of the CS blending process (Fig. 1B).

#### Fourier-transform infrared spectroscopy (FTIR)

The dominant crystalline structures of SF can be divided into two types: (i) the type Silk I molecular chains are formed from an alternate accumulation of the α-helix and the β-parallel fold conformation; (ii) the type Silk II molecular chains are layered structures of the anti-parallel β-sheet, which favors structural stabilization [21, 32]. Thus, transforming random coil or helical conformation into β-sheet structure favors the structural stability of SF products. As shown in Fig. 1C, the scaffolds displayed intense absorption peaks around 1632 cm^−1^, 1530 cm^−1^, and 1235 cm^−1^, which characterized the Silk II structure. Consistent with previous reports, genipin and CS significantly contributed to the formation of β-sheet structure in SF [23, 33]. Additionally, the broad peak from 1000 to 1100 cm^−1^ corresponded to the Si–O stretch and was a characteristic peak of CS, which strengthened in proportion to the CS content [34].

#### XRD

XRD spectra (Fig. 1D) showed that CS had diffraction peaks around 2θ = 26°, 27°, 29° and 33°. As the CS content increased, these peaks became more prominent, indicating that more CS was incorporated into our 3D-printed scaffolds. These results were consistent with the EDS and FTIR spectra above.

#### Swelling ratio, water absorption and porosity

Figure 1E, F presented the swelling ratios and water absorption of 3D-printed scaffolds. Compared with the SG group, the addition of CS reduced the swelling ratio and water absorption of the hydrogel scaffolds. This reduction may be due to the incorporation of the low-swelling material (CS) into the SF and gelatin network. However, even with the addition of CS, the swelling ratio and water absorption of the scaffolds were still high enough to ensure infiltration of cells and transport of nutrients [35].

The porosity of the composite scaffolds with or without CS had no significant differences and was all higher than 65% (Fig. 1G). This level of porosity was high enough to allow for the exchange of multiple nutrients required for bone formation [36]. Therefore, the porosity of these scaffolds prepared in this study was appropriate for bone regeneration.

#### Mechanical properties

As illustrated in Fig. 1H–I, the stress–strain curve and the compression modulus of scaffolds under compressive process were analyzed. Compared to SG scaffolds (3.377 ± 0.6536 kPa), the compressive modulus of the scaffolds increased from 14.78 ± 0.6108 kPa to 57.09 ± 5.556 kPa with the increase of CS content from the SGC_L_ group to the SGC_H_ group, while there were no significant differences between the SGC_L_ group and the SGC_M_ group. Previous studies have shown that mesenchymal stem cells (MSCs) tend to differentiate towards osteogenic lineage in a microenvironment with 11–30 kPa stiffness [37]. Therefore, the compressive modulus of the SGC_L_ group and the SGC_M_ group were 14.78 ± 0.6108 kPa and 18.2 ± 2.339 kPa, respectively, which were suitable for the osteogenic differentiation of MSCs.

#### Weight loss

Figure 1J and Additional file 1: Fig. S2 illustrated the weight loss of the scaffolds after immersion in simulated body fluid (SBF) for 21 days. We could observe that there was no noteworthy variation in the rate of deterioration among the four groups during the initial 7 days. By day 14, the degradation rate of the SG group accelerated, while the SGC_M_ group exhibited the slowest degradation among the four groups. By the 21st day, the SG group continued to display the quickest degradation rate, and there were notable variations in the degradation rates among the three CS-added scaffolds. Specifically, the SGC_L_ group exhibited faster degradation rate compared to the SGC_H_ group, while the SGC_M_ group exhibited the slowest degradation rate, probably due to its appropriate powder-to-liquid ratio. In addition, we also performed SEM examination on the samples after 7 days of immersion and found that none of the four groups had significant collapse in their general morphology, as shown in Additional file 1: Fig. S3. Meanwhile, we could clearly see that the surface of the SG group became the most porous, followed by the SGC_L_ and SGC_H_ groups, while the surface morphology of the SGC_M_ group did not change much, which was consistent with the subsequent degradation ratio of the SG and SGC_M_ groups (which are the fastest and slowest among the four groups).

It is well known that bone repair takes a long time, so biomaterials applied in this field need a suitable degradation ratio to match the rate of bone regeneration [38]. Hence, the addition of CS enabled the 3D-printed hydrogel scaffold to degrade at a rate more similar to bone regeneration, which may enhance the healing process.

#### In vitro biomineralization

Currently, the hydroxyapatite (HA) deposition rate on the surfaces of samples soaked in SBF is considered a valid in vitro method for assessing the biomineralization capacity of biomaterials [39]. Following a 7-day immersion of the scaffolds in SBF, HA precipitation was noted on all groups' surfaces, except for the SG group. It is worth noting that the apatite spherulites deposition was promoted by the increase in CS content. The EDS spectra indicated Ca/P ratios of approximately 5.60, 2.16, and 1.76 in the SGC_L_, SGC_M_, and SGC_H_ groups, respectively (Additional file 1: Fig. S4). These results demonstrated that elevated CS content resulted in improved HA deposition, as confirmed by SEM photographs. As previously reported, the presence of CS may be able to encouraged the deposition of HA due to its interaction with SBF [40, 41].

### Cell viability and adhesion

**Fig. 2:**
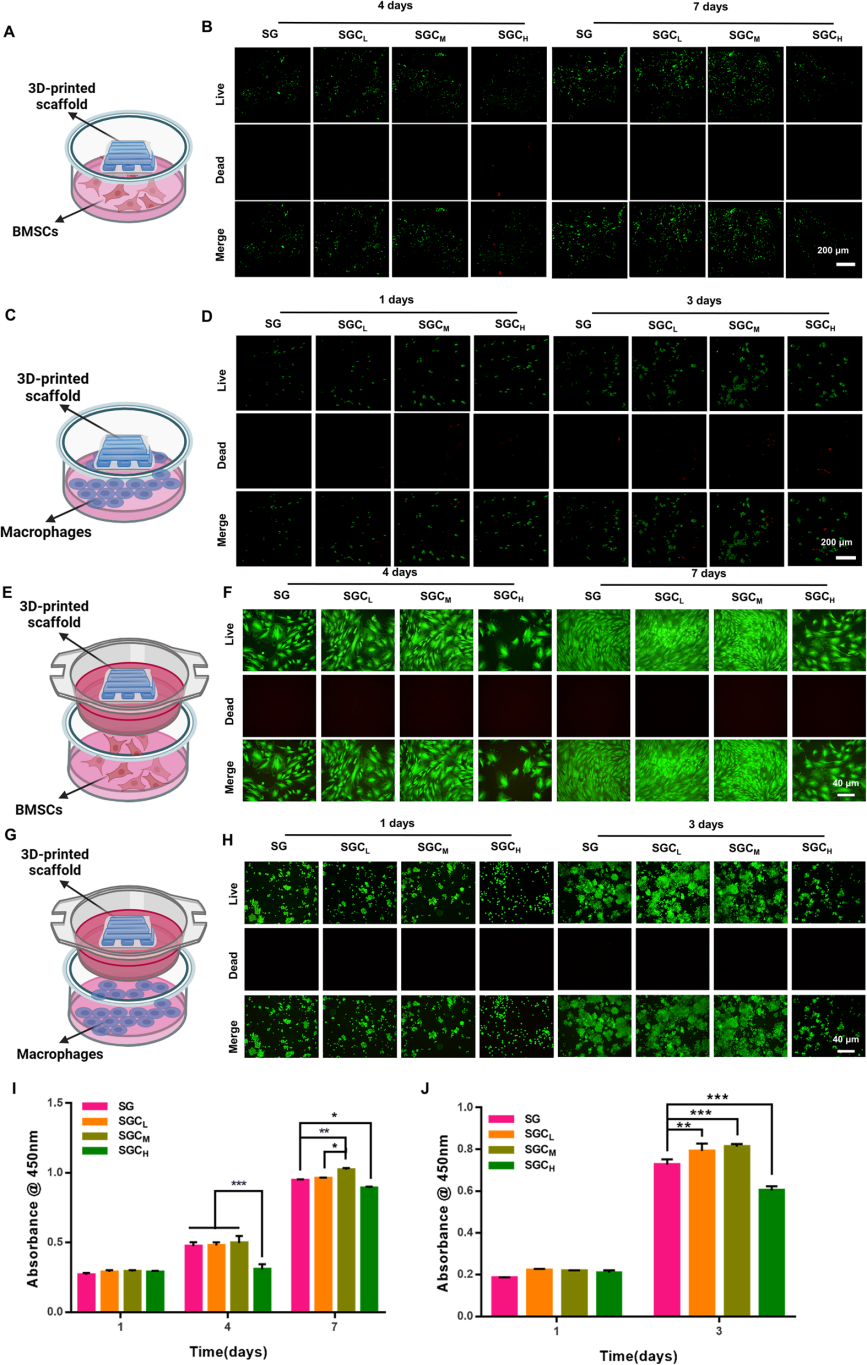
Cell viability of the 3D-printed scaffolds on BMSCs and macrophages. **A**, **C** Schematic illustration of the co-culture models- "co-culture on" hydrogels. **B**, **D** The Calcein-AM/PI staining of BMSCs and macrophages co-cultured on SG, SGC_L_, SGC_M_ and SGC_H_ scaffolds. **E**, **G** Schematic illustration of the co-culture models- "co-culture with" hydrogels. **F**, **H** The Calcein-AM/PI staining of BMSCs and macrophages co-cultured with SG, SGC_L_, SGC_M_ and SGC_H_ scaffolds. **I** CCK-8 assay of BMSCs after cultured for 1, 4, and 7 days.** J** CCK-8 assay of macrophages after cultured for 1 and 3 days. ^*^P < 0.05, ^**^P < 0.01 or.^***^P < 0.001

Prior to determining the efficacy of using scaffolds for bone repair, it was necessary to confirm their impact on cell viability. To evaluate hydrogel cell viability, we utilized two previously reported cell-hydrogel co-culture models- ”co-culture on” and “co-culture with” hydrogels [42]. Cell viability was assessed using the calcein-AM/propidium iodide (PI) double staining kit and cell counting kit-8 (CCK-8). The results indicated that there was no significant decrease in cell viability for either BMSCs or macrophages after 1 day of incubation. However, on days 4 and 7 of BMSCs culture and day 3 of macrophage culture, the SGC_H_ group resulted in inhibited cell viability, while the other groups exhibited excellent biocompatibility for BMSCs and macrophages at these time periods (Fig. 2A–J).

Cell adhesion has been found to play a crucial role in the differentiation of MSCs, while a spreading morphology has been observed to facilitate osteogenesis [43, 44]. Confocal laser scanning microscope (CLSM) demonstrated that BMSCs co-cultured on scaffolds extended polygon shape with obvious pseudopodia on the SG, SGC_L_ and SGC_M_ groups after cultured for 4 and 7 days. Nevertheless, the SGC_H_ group had a significant inhibitory effect on cell spreading and proliferation of BMSCs (Additional file 1: Fig. S5A). Additionally, the trend observed in the F-actin/DAPI staining of BMSCs co-cultured with scaffolds was consistent with the results of the “co-culture on” method (Additional file 1: Fig. S5B,C). Based on the above findings, the SGC_H_ scaffold was excluded due to its obvious cytotoxicity on BMSCs and macrophages, and SG, SGC_L_ and SGC_M_ scaffolds were selected for further experiments.

### BMSCs osteo-/angiogenic differentiation

As vascularization plays a crucial role in the healing of bone defects, the assessment of biomaterials for bone repair should take into account not only their osteoinductive function but also their pro-angiogenic function [45, 46]. To evaluate the above key indicators, we employed quantitative real-time polymerase chain reaction (qRT-PCR) technology to examine the osteogenic genes such as bone morphogenetic protein-2 (BMP-2), collagen type-1 (COL-1), osteocalcin (OCN), osteopontin (OPN), osteonectin and runt-related transcription factor-2 (RUNX-2), as well as angiogenic genes such as basic fibroblast growth factor (bFGF) and vascular endothelial growth factor (VEGF) of BMSCs.

**Fig. 3:**
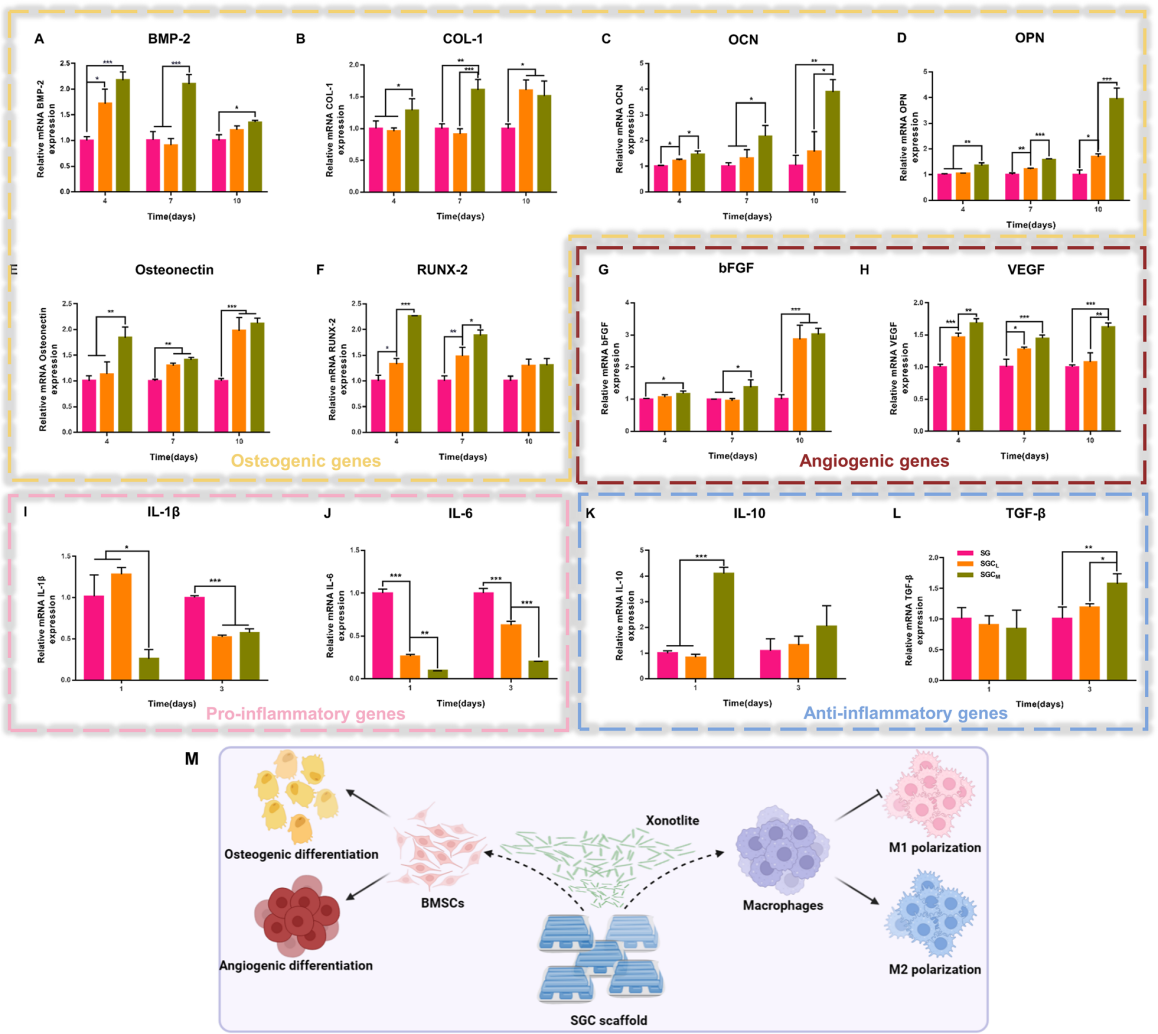
The effect of the 3D-printed scaffolds on BMSCs osteo-/angiogenesis and macrophage reprogramming, **A–H** qRT-PCR analysis in BMSCs of osteogenesis-related genes (BMP-2, COL-1, OCN, OPN, Osteonectin and RUNX-2) and angiogenesis-related genes (bFGF and VEGF) after cultured for 4, 7, and 10 days. **I–L** qRT-PCR analysis in macrophages of pro-inflammatory genes (IL-1β and IL-6) and anti-inflammatory genes (IL-10 and TGF-β) after cultured for 1 and 3 days. **M** Schematic illustration of the effects of SGC scaffold on osteo-/angiogenesis of BMSCs and polarization of macrophages. ^*^P < 0.05, ^**^P < 0.01 or.^***^P < 0.001

The results presented in Fig. 3A–H demonstrated that BMSCs cultured with CS-containing scaffolds effectively promoted their osteo-/angiogenic capacity in comparison to the SG group. The production of COL-1 (day 10), osteonectin (day 7 and 10) and bFGF (day 10) was promoted by CS-containing groups, whereas no significant differences were observed between the SGC_L_ and SGC_M_ groups. Additionally, the mRNA expression of RUNX-2 (day 10) did not differ significantly among the three groups. Apart from the genes mentioned at the corresponding time points above, the SGC_M_ group exhibited the strongest promotion of osteo-/angiogenic gene expression. CS has been shown to be efficacious in improving BMSCs osteo-/angiogenic differentiation by virtue of the bioactive ions it releases, which is consistent with our study [1]. In addition, we identified OPN expression of osteogenesis-related proteins by immunofluorescence staining, and its trend was consistent with the previous results of gene expression (Additional file 1: Fig. S6A, B).

### Macrophage reprogramming

Macrophages are among the initial cells to infiltrate the regions where bone defects have occurred, and their contribution is essential for successful bone rejuvenation. Researches have demonstrated that the constant presence of M1phenotype macrophages prolonged the local pro-inflammatory microenvironment, leading to biomaterial implantation failure and impaired bone healing. In contrast, M2 phenotype macrophages promoted an anti-inflammatory microenvironment that was conducive to osteo-/angiogenesis [47]. Therefore, the implanted biomaterials that induce macrophage polarization towards M2 phenotype are essential for the local bone repair [48].

The qRT-PCR results presented in Fig. 3I–L demonstrated that RAW264.7 macrophages underwent reprogramming after 1 or 3 days of culture when exposed to scaffolds with CS. The CS supplementation significantly reduced the expression of M1 phenotype macrophage markers (IL-1β and IL-6) compared to the SG group (day 10), with the strongest inhibition in the SGC_M_ group. In addition, compared to the SG group, the SGC_L_ group containing a minimal amount of CS did not significantly affect the expression level of M2 macrophage markers, while the SGC_M_ group containing a higher amount of CS increased the expression level of M2 macrophage markers including IL-10 (day 1) and TGF-β (day 3) significantly. These data suggested that CS incorporation not only effectively inhibited macrophages' M1 polarization but also promoted their conversion to the M2 phenotype at a suitable addition level. The immunomodulatory function of CS may be due to the release of biologically active Si ions, as previously reported to have anti-inflammatory properties [25].

Taken together, the addition of CS provided excellent osteo-/angiogenic and immunomodulatory properties to the SG scaffold (Fig. 3M). And the optimal addition of CS was observed in the SGC_M_ group due to its prominent ability to induce osteo-/angiogenesis and reprogram macrophage towards M2 polarization. Thus, we selected the SGC_M_ group to conduct the follow-up study.

### The influence of macrophage-derived conditioned mediums (CMs) on the migration and the osteo-/angiogenic differentiation of BMSCs

**Fig. 4:**
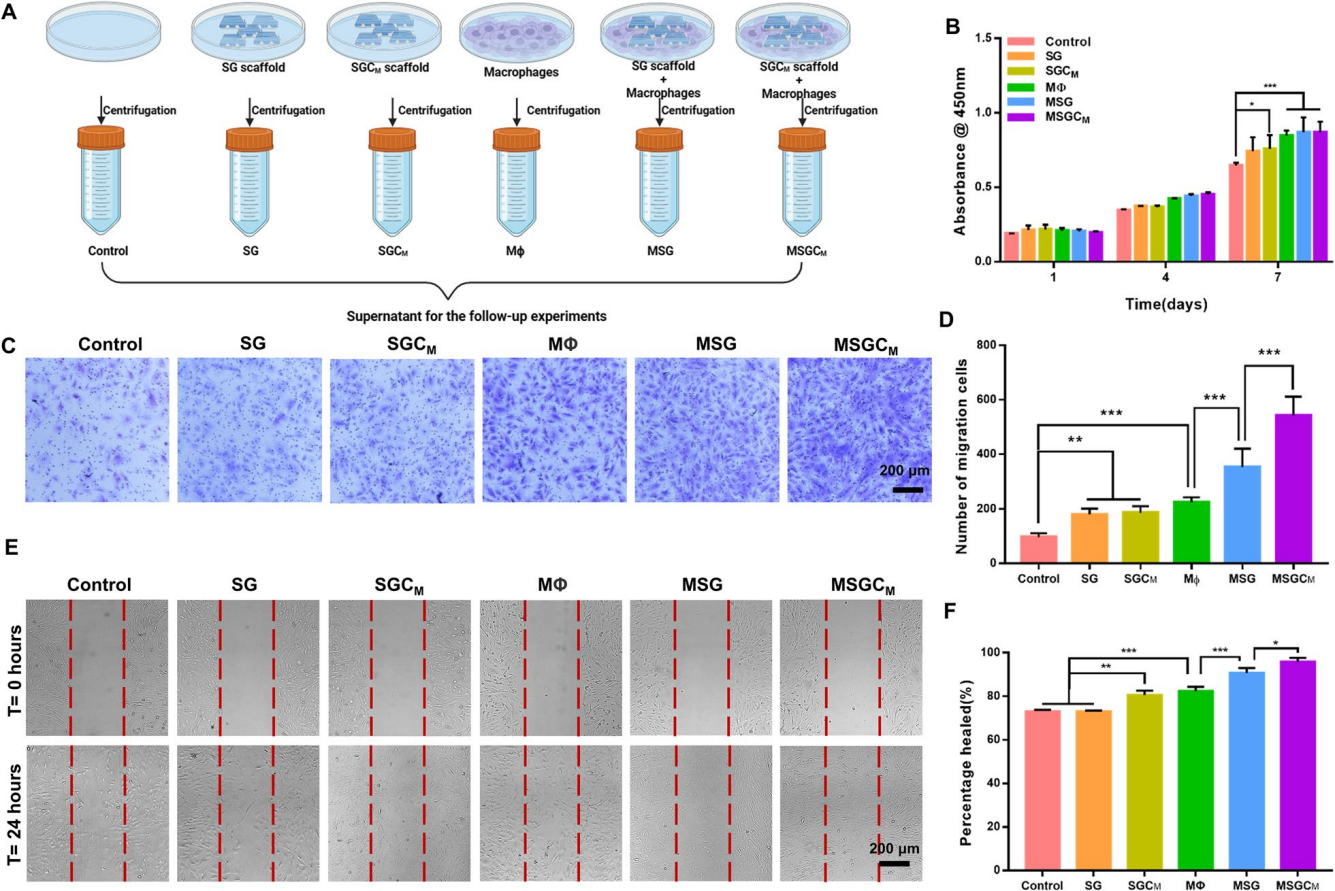
The influence of CMs on the migration of BMSCs. **A** Schematic illustration of the preparation process and the specific grouping of the macrophage derived CMs.** B** Effect of CMs on viability of BMSCs. **C**, **D** The crystal violet staining and quantitative analysis of transwell assay for BMSCs cultured in CMs. **E**, **F** The wound healing assay and its quantitative assay for BMSCs cultured in CMs. ^*^P < 0.05, ^**^P < 0.01 or.^***^P < 0.001

Successful bone regeneration requires enough recruitment and robust osteo-/angiogenic differentiation of BMSCs, which are not only regulated by the surface properties of the implant biomaterials but also by the surrounding osteoimmune microenvironment [49]. Therefore, we conducted further investigation into the effects of the SGC_M_-influenced osteoimmune microenvironment on the regulation of in vitro recruitment and osteo-/angiogenic differentiation of BMSCs using macrophage-derived CMs. Figure 4A depicted the preparation process and the specific grouping of the CMs.

#### Migration of BMSCs with CMs

Initially, it was necessary to determine whether the CMs had any adverse effects on the cell viability of BMSCs. On the 1st and 4th day, the cell viability showed no notable differences among all groups. Moreover, on the 7th day, BMSCs co-cultured with CMs derived from macrophages exhibited a notable increase in comparison to the control, SG, and SGC_M_ groups, while there was no comparability among MΦ, MSG and MSGC_M_ groups (Fig. 4B). These data indicated that BMSCs survived well in all CMs’ groups.

Since host MSCs' infiltration and recruitment to the impairment site are crucial factors for bone repair, we used the transwell assay and the scratch assay in vitro to assess BMSCs' migratory ability [13]. According to the images from the transwell assay, the number of BMSCs that vertically migrated to the lower chambers significantly increased in all groups except the control group (Fig. 4C). Moreover, quantitative analysis further indicated that macrophage-derived CMs attracted more BMSCs to the lower surface than control, SG and SGC_M_ groups, while the number of BMSCs migrating to the lower chambers in the MSG and MSGC_M_ groups was ∼1.6- and ∼2.4-fold higher than that in the MΦ group.

Figure 4E, F displayed the horizontal migratory ability of the cells in all groups. The MSGC_M_ group demonstrated the greatest efficiency in enhancing the horizontal migration of BMSCs, followed by the MSG group, and then the SGC_M_ and MΦ groups. Among them, there was no statistically significant difference between the SGC_M_ group and MΦ groups, both of which were stronger than the control and SG groups.

The above findings suggested that the incorporation of CS not only enhanced the direct pro-migration ability of BMSCs slightly more in the SGC_M_ group than in the SG group, but also indirectly contributed to an evident increased BMSCs’ recruitment ability via mediation of the macrophage-regulated osteoimmune microenvironment.

#### Osteo-/angiogenic differentiation of BMSCs with CMs

**Fig. 5:**
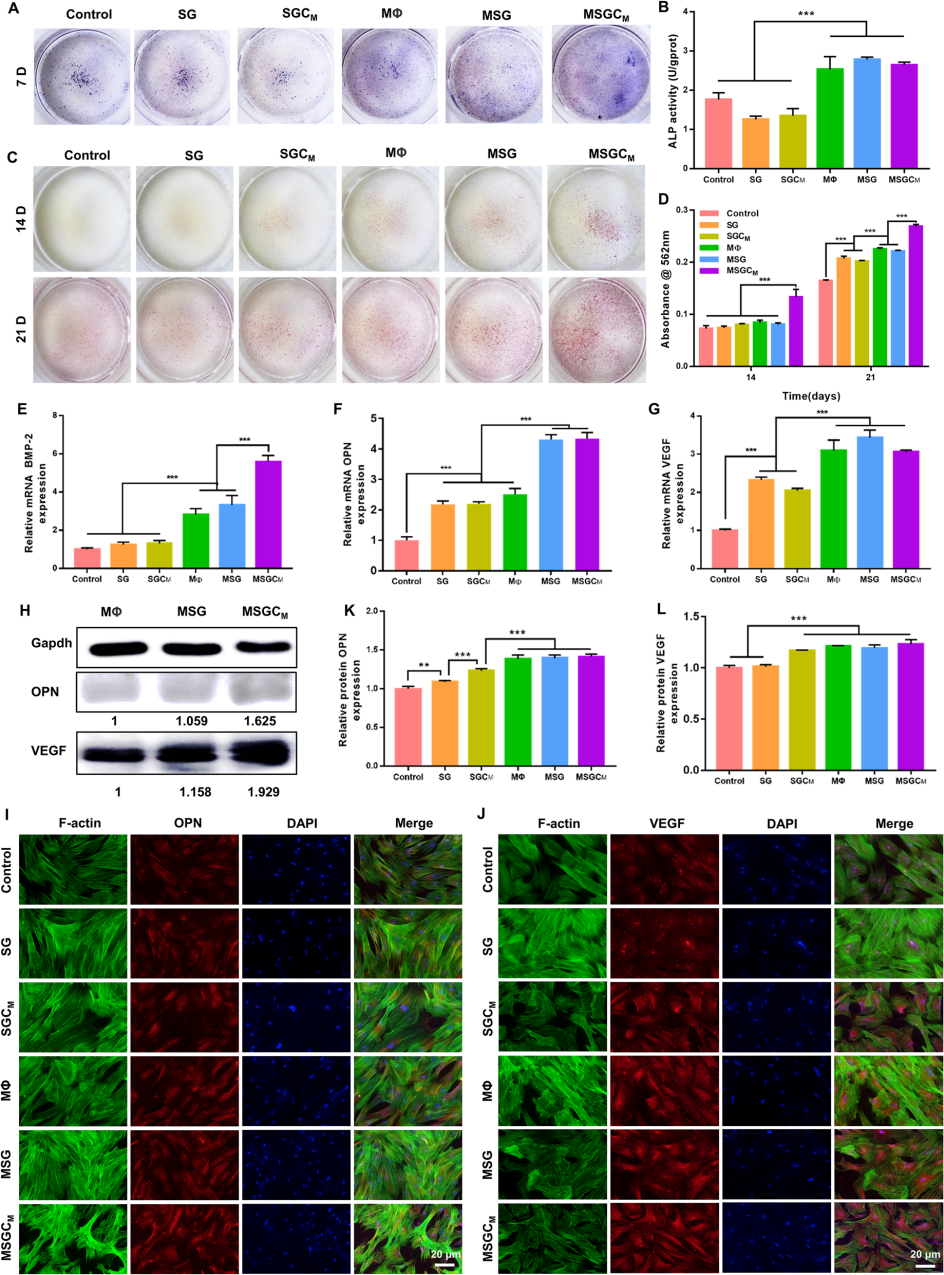
The influence of CMs on the osteo-/angiogenic differentiation of BMSCs. **A**, **B** The ALP staining images and its quantitative assay of BMSCs cultured on CMs for 7 days. **C**, **D** The ARS staining and its quantitative assay of BMSCs cultured on CMs for 14 and 21 days. **E–G** qRT-PCR analysis of BMP-2, OPN and VEGF mRNA expression in BMSCs cultured on CMs for 7 days. **H** The western blot of the OPN and VEGF e protein expression in BMSCs cultured on CMs for 7 days. **I–L** The immunofluorescence staining and quantitative assay of the OPN and VEGF protein in BMSCs cultured on CMs for 14 days. ^*^P < 0.05, ^**^P < 0.01 or.^***^P < 0.001

The results of alkaline phosphatase (ALP) staining and ALP activity assay, conducted after 7 days of cultivation of BMSCs in various CMs, demonstrated that the groups cultured with macrophage-derived CMs had significantly higher ALP levels than other groups, while there was no notable difference between the MΦ, MSG and MSGC_M_ groups (Fig. 5A, B). Alizarin red S (ARS) staining was used to demonstrate the BMSCs’ mineralization capacity on days 14 and 21 (Fig. 5C). Among all groups, the MSGC_M_ group presented the most demonstrable mineralization. Quantitative analysis of ARS staining further indicated that the mineralization effect of MSGC_M_ group outperformed other groups in terms of promoting calcium deposition (Fig. 5D). To sum up, MSGC_M_ group possessed strongest capacity for promoting calcium deposition. In addition, qRT-PCR analysis showed that after 7 days of culture, BMSCs in the MSG and MSGC_M_ groups had stronger promotion of osteo-/angiogenic gene expression compared to BMSCs in other groups (Fig. 5E–G). In particular, the MSGC_M_ group resulted in higher expression of BMP-2 than the MSG group, although there was no significant difference in OPN and VEGF expression between the two groups. Western blot analysis further indicated that that MSGC_M_ group had the strongest effect on promoting the OPN and VEGF protein expression (Fig. 5H). Also, immunofluorescence staining and its quantitative results (Fig. 5I–L) showed that OPN and VEGF protein expression in the MΦ, MSG and MSGC_M_ groups was higher than those in other groups.

As known to all, macrophages were pivotal in bone repair by secreting multiple cytokines [50]. IL-10 and TGF-β secreted by M2 macrophages have been helpful in cell migration, bone reconstruction and vascularization [51, 52]. Furthermore, CS to some extent as a bio-ceramic could promote the attachment, proliferation and osteo-/angiogenic differentiation of BMSCs [53]. Therefore, the upregulated migratory capability and osteo-/angiogenic differentiation capacity of BMSCs incubated in MSGC_M_ may be related to the increased expression of IL-10 and TGF-β as well as the release of Ca and Si ions of CS.

### In vivo study

The above in vitro studies have demonstrated that the SGC_M_ group could directly promote BMSCs’ osteo-/angiogenesis and reprogram macrophages towards M2 polarization, as well as also indirectly drive remodeling of the optimal osteoimmune microenvironment to stimulate bone regeneration. These positive results have inspired us to conduct further research on their capacity to trigger bone formation and restructure the osteoimmune microenvironment in vivo.

#### Micro-CT analysis

**Fig. 6:**
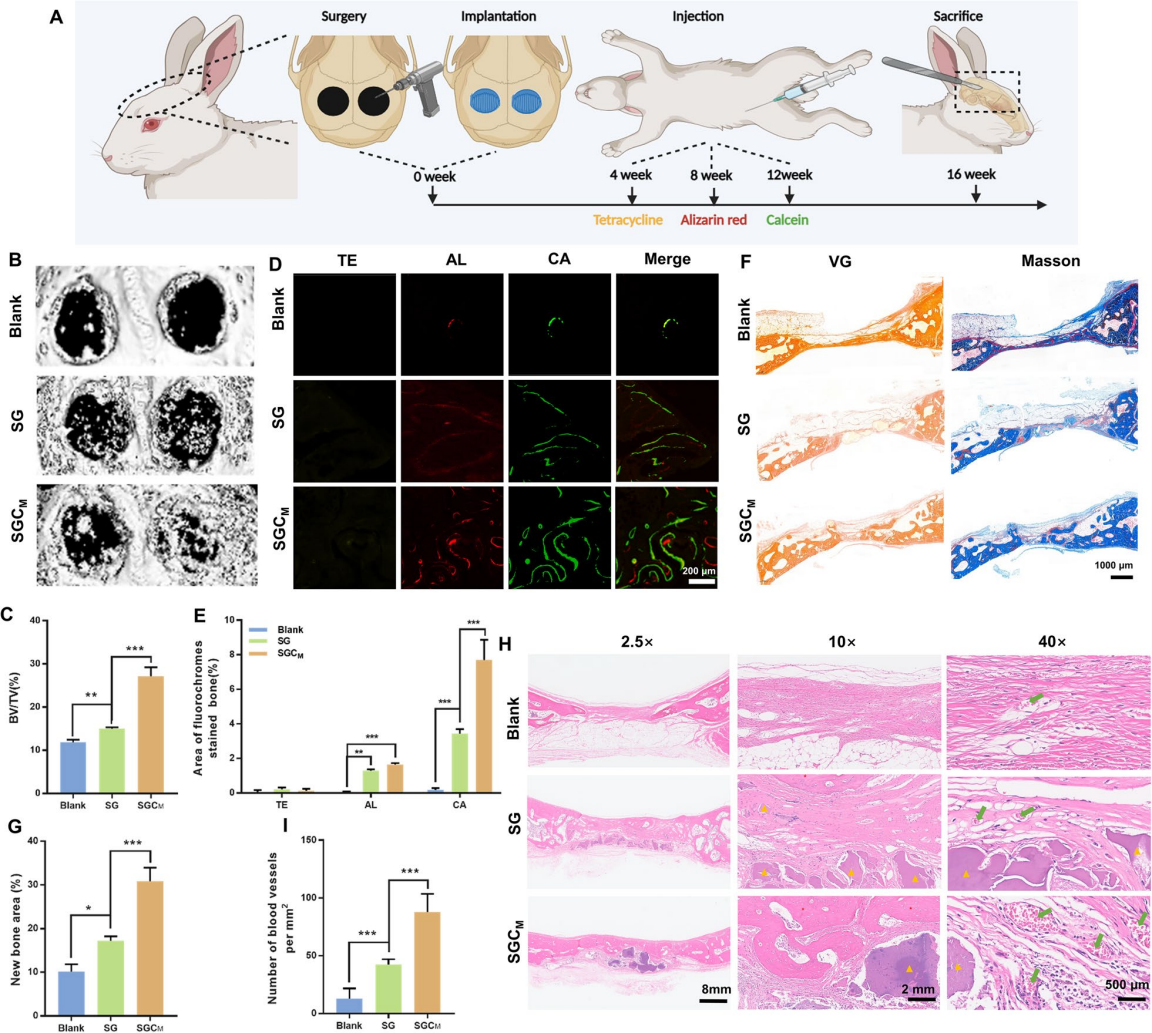
In vivo evaluation of the 3D-printed scaffolds on vascularized bone regeneration. **A** Schematic illustration of the surgery procedure. **B** Micro-CT 3D-reconstructed images of the calvarial defects. **C** Measurement of BV/TV. **D** Sequence fluorescence staining images of the calvarial defects. **E** Percentage of fluorescence labeled regions in D**. F** Representative photographs of the VG and Masson staining in the calvarial defect areas. **G** Quantification of the new bone area in F. **H** H&E staining the calvarial defect areas (the red* denotes the newly formed bone, the yellow ▲ marks the residual biomaterial, and the green arrow marks the capillaries). **I** The number of newly formed blood vessels in H. ^*^P < 0.05, ^**^P < 0.01 or.^***^P < 0.001

The bone repair capability of 3D-printed scaffolds was evaluated through implantation in the rabbit calvarial defect model as depicted in Fig. 6A. The Micro-CT 3D-reconstructed models of the calvarial defects illustrated that the SGC_M_ group had the strongest ability for new bone formation among the three groups (Fig. 6B). Moreover, the result of bone volume/total volume ratio (BV/TV) value analysis was consistent with the result shown in Fig. 6C.

#### Sequential fluorescent labeling

Figure 6D, E displayed sequential fluorescence labeling images and their quantitative analysis at week 4 (Tetracycline hydrochloride (TE), yellow), 8 (alizarin red (AL), red), and 12 (calcitonin (CA), green) post-modeling. New bone formation mainly took place during the late stage in all three groups, with little to no occurrence during the early stage. The SGC_M_ group showed the highest fluorescence regions of AL and CA among the three groups, whereas the TE fluorescence region did not show any significant difference among three groups. The above results suggested that the SGC_M_ group with added CS had a more effective ability to promote new bone formation in vivo compared to the SG group, and its efficacy primarily took place during the middle and late stages of osteogenesis.

#### Van gieson (VG) staining and masson's trichrome staining

Figure 6F, G demonstrated that the SGC_M_ group exhibited a significant increase in the area of newly formed bone in comparison to the blank and SG group. Analysis of the VG staining and masson's trichrome staining results of undecalcified samples indicated consistent results with the those of Micro-CT analysis and sequential fluorescent labeling.

#### Haematoxylin and eosin (H & E) staining

Tissue specimens were decalcified and sectioned for H&E staining at 16 weeks postoperatively. The H&E images revealed small areas of new bone formation and neovascularization at the edge of the defect in the blank group, causing the overlying fibrous tissue to collapse into the defect. Furthermore, bone regeneration was stronger in both the SG and SGC_M_ groups when compared to the blank group, suggesting that the transplantation of biomaterials promoted tissue reparation. Of the three groups, the SGC_M_ group presented the richest structures of bone trabeculae and capillaries (Fig. 6I).

#### Immunohistochemical staining

**Fig. 7:**
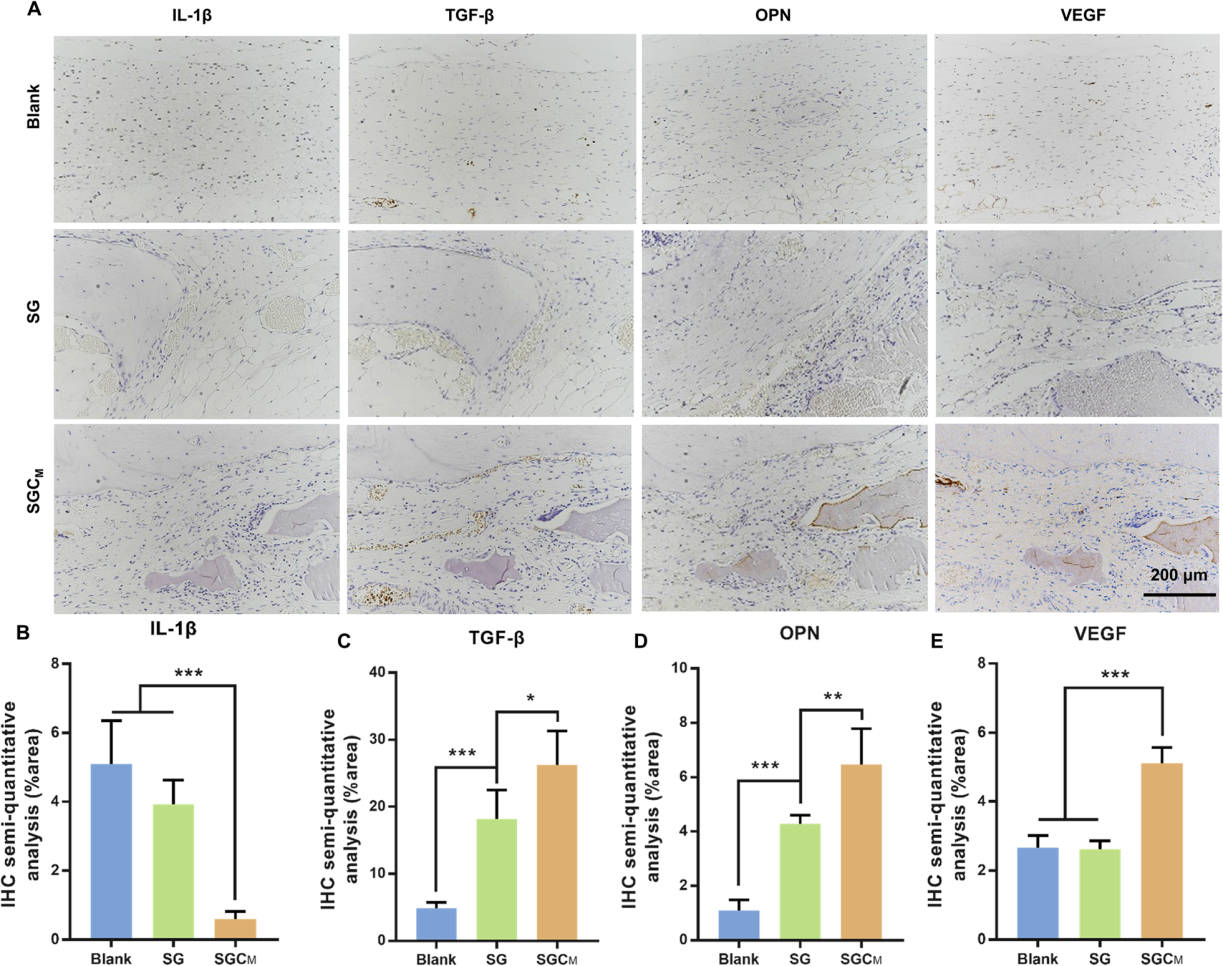
In vivo immunohistochemistry evaluation of the 3D-printed scaffolds on vascularized bone regeneration. **A** Immunohistochemistry staining of IL-1β, TGF-β, OPN and VEGF. **B–E** Semi-quantitative evaluation of the relative expression of IL-1β, TGF-β, OPN and VEGF in the three groups according to **A**. ^*^P < 0.05, ^**^P < 0.01 or.^***^P < 0.001

Immunohistochemical staining and its semi-quantitative analysis of decalcified skulls for the inflammatory marker (IL-1β), anti-inflammatory marker (TGF-β), osteogenic marker (OPN) and angiogenic marker (VEGF) were used to investigate the in vivo situation regarding osteo-/angiogenesis and macrophage polarization (Fig. 7A–E). The SGC_M_ group induced higher productions of TGF-β, OPN and VEGF than SG group, while these productions were weakest in the blank group (Fig. 7A, C–E). However, the expression level of IL-1β was notably downregulated in the SGC_M_ group (Fig. 7A, B).

In recent years, growing evidence showed that suitable biomaterials contributing to bone regeneration should not only aid in bone repair, but also modulate the immune response to remodel suitable osteoimmune microenvironment [54]. Results from the above experiment demonstrated that the incorporation of CS can notably improve bone regeneration and blood vessel formation as well as effectively manage osteoimmune microenvironment in vivo.

### Analysis of immune regulation mechanism of SGCM based on RNA sequencing

To identify the potential immunomodulatory mechanisms in the SGC_M_ hydrogel, RNA sequencing was performed to determine the differentially expressed genes (DGEs) in RAW264.7 cultured in control and SGC_M_ groups for 3 days.

**Fig. 8:**
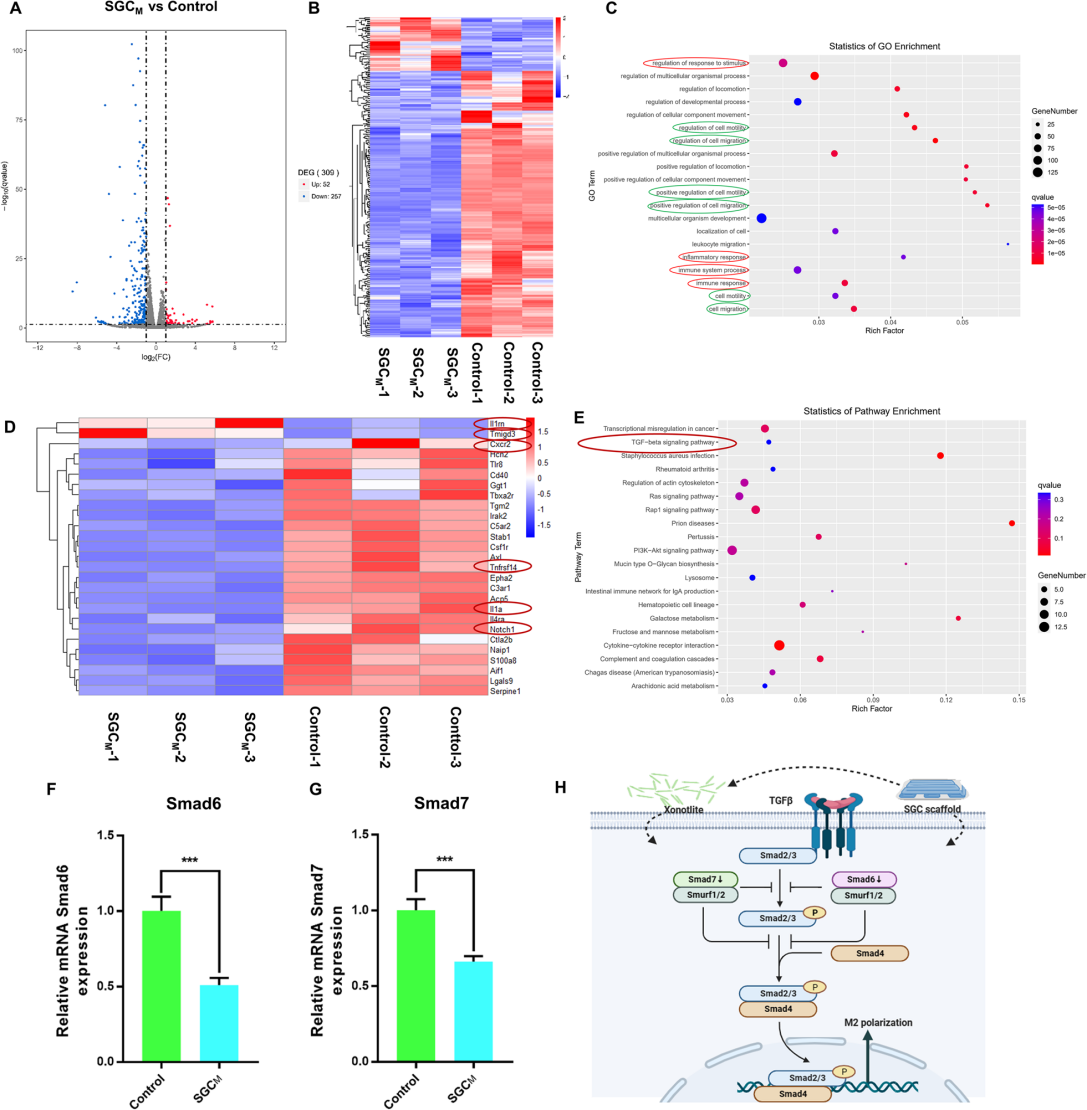
The mechanism of the 3D-printed scaffolds on macrophage reprogramming. **A**, **B** The volcano map and hot map of RAW264.7 cultured in control and SGC_M_ groups showing the different gene expression. **C** GO enrichment between control and SGC_M_ groups (red circles represent immune-related processes green circles represent migration-related processes). **D** Hot map of DEGs about immune and inflammation. **E** KEGG pathway gene enrichment analysis. **F**, **G** mRNA expression of Smad 6 and Smad 7 which involved in TGF-β pathway. **H** Schematic illustration of the mechanisms of SGC_M_ on macrophage reprogramming. ^*^P < 0.05, ^**^P < 0.01 or.^***^P < 0.001

Figure 8A, B shows the volcano and heat maps created to depict the DEGs between control and SGC_M_ groups in RAW264.7. There were 52 upregulated genes and 257 downregulated genes. In the gene ontology (GO) biological process (BP) category, the immune-related processes (immune system process, inflammatory response, regulation of response to stimulus and immune response) and migration-related processes (regulation of cell motility, regulation of cell migration, positive regulation of cell motility, positive regulation of cell migration, cell motility and cell migration) were clearly enriched (Fig. 8C). The function mentioned above helped to make SGC_M_ scaffold exercise its anti-inflammatory and pro-migratory effects.

Based on the results of the BP of GO analysis, we selected the genes related to inflammation and immune processes to draw the heatmap and found the anti-inflammatory genes such as Il1RN [55–57] and Tmigd3 [58] were significantly upregulated in group SGC_M_, while the pro-inflammatory genes such as cxcr2 [59], Il1a [57], Notch1 [60], Tnfrsf14 [61] and etc. [62] were evidently downregulated in the SGC_M_ group (Fig. 8D). The findings above confirm our previous conclusion on the SGC_M_ hydrogel's macrophage reprogramming function towards M2 polarization. Kyoto Encyclopedia of Genes and Genomes (KEGG) analysis shown in Fig. 8E present 20 notably enriched pathways related to immunomodulation, including the Rap1 signaling pathway (mmu04015), PI3K-Akt signaling pathway (mmu04151), Ras signaling pathway (mmu04014), Rheumatoid arthritis (mmu05323) and TGF-β signaling pathway (mmu04350). Combined with the DEGs, Smad6 and Smad7 as important inhibitory molecules in the TGF-β cascade have already been proved to be able to regulate the inflammatory response in macrophage [63]. Activation of the TGF-β/Smad signaling pathway has been reported to promote M2 polarization of macrophages [64]. However, the overexpression of Smad6 and Smad7 also recruits Smad ubiquitination regulatory factor 1(Smurf1) and Smurf2 as well as inhibits receptor-associated Smads (R-Smads) binding to receptors, phosphorylation, and polymerization with Smad4 [65].Therefore, we used qRT-PCR to verify the mechanism of TGF-β signaling pathway in anti-inflammatory function of SGC_M_ scaffold. From Fig. 8F–G, Smad6 and Smad7 were clearly decreased in SGC_M_ group, which further demonstrated that SGC_M_ hydrogel may exert its anti-inflammatory effects through decreasing Smad6 and Smad7. Collectively, the mechanism of macrophage reprogramming by the 3D-printed SGC_M_ composite hydrogel scaffold was shown in Fig. 8H below, which may be achieved by the surface of the material or by the CS released from the material.

## Conclusion

Our study focused on the development of a 3D-printed composite hydrogel scaffold (SGC) that coordinated with both osteo-/angiogenesis and osteoimmune microenvironment. The CS released from this 3D-printed composite scaffold endowed the SGC scaffold with various functions, such as promoting osteo-/angiogenic differentiation of BMSCs, reprogramming macrophages into M2 phenotype via inhibiting Smad6 and Smad7, and remodeling a favorable osteoimmune microenvironment conducive to osteo-/angiogenesis. Furthermore, by using a rabbit calvarial defect model, we demonstrated that the SGC scaffold promoted in situ vascularized bone regeneration through osteoinduction and osteoimmune modulation. Overall, the SGC scaffold-mediated robust osteoinductivity and favorable osteoimmunomodulatory properties are considered a promising strategy for the treatment of complicated bone defects.

### Supplementary Information

**Additional file 1: Table S1.** The sequences of BMSCs genes (F: forward primer R: reverse primer). **Figure S1.** **A**, **B** XRD and TEM of CS. **C** The Viscosity-shear rate curve of SG, SGC_L_, SGC_M_ and SGC_H_. **Figure S2.** The degradation ratio of SG, SGC_L_, SGC_M_ and SGC_H_ samples in histogram.^*^P < 0.05,^**^P < 0.01 or ^***^P < 0.001. **Figure S3.** SEM photographs in low magnification lens of SG, SGC_L_, SGC_M_ and SGC_H_ sampled after degradation for 7 days. **Figure S4.** SEM photographs in high magnification lens and EDS analysis of composite specimens with different CS contents immersed in SBF (yellow arrow marks the apatite spherulites). **Figure S5.** **A** The F-actin/DAPI staining of BMSCs “co-culture on” SG, SGC_L_, SGC_M_ and SGC_H_ scaffolds. **B** Schematic illustration of the co-culture models-“co-culture with” hydrogels. **C** The F-actin/DAPI staining of BMSCs “co-culture with” SG, SGC_L_, SGC_M_ and SGC_H_ scaffolds. **Figure S6.** **A**, **B** The immunofluorescence staining and its quantitative assay of the OPN protein in BMSCs after cultured for 7 days. ^*^P < 0.05, ^**^P < 0.01 or ^***^P < 0.001.

## Data Availability

The datasets used and/or analyzed during the current study are available from the corresponding author on reasonable request.
