# Supplementary material for: A xonotlite nanofiber bioactive 3D-printed hydrogel scaffold based on osteo-/angiogenesis and osteoimmune microenvironment remodeling accelerates vascularized bone regeneration
Source: J Nanobiotechnology. 2024 Feb 12;22:59. doi: 10.1186/s12951-024-02323-9 (PMC10863132; doi:10.1186/s12951-024-02323-9)
Supplement: Supplementary file 1 — Additional file 1: Table S1. The sequences of BMSCs genes (F: forward primer R: reverse primer). Figure S1. A, B XRD and TEM of CS. C The Viscosity-shear rate curve of SG, SGCL, SGCM and SGCH. Figure S2. The degradation ratio of SG, SGCL, SGCM and SGCH samples in histogram.*P < 0.05,**P < 0.01 or ***P < 0.001. Figure S3. SEM photographs in low magnification lens of SG, SGCL, SGCM and SGCH sampled after degradation for 7 days. Figure S4. SEM photographs in high magnification lens and EDS analysis of composite specimens with different CS contents immersed in SBF (yellow arrow marks the apatite spherulites). Figure S5. A The F-actin/DAPI staining of BMSCs “co-culture on” SG, SGCL, SGCM and SGCH scaffolds. B Schematic illustration of the co-culture models-“co-culture with” hydrogels. C The F-actin/DAPI staining of BMSCs “co-culture with” SG, SGCL, SGCM and SGCH scaffolds. Figure S6. A, B The immunofluorescence staining and its quantitative assay of the OPN protein in BMSCs after cultured for 7 days. *P < 0.05, **P < 0.01 or ***P < 0.001. [file 12951_2024_2323_MOESM1_ESM.docx]

**Additional file 1**

**A xonotlite nanofiber bioactive 3D-printed hydrogel scaffold based on osteo-/angiogenesis and osteoimmune microenvironment remodeling accelerates vascularized bone regeneration**

Shi-Yuan Yang^a, b,1^, Yu-Ning Zhou ^a, b,1^, Xing-Ge Yu ^b, c,1^, Ze-Yu Fu ^b, c^, Can-Can Zhao ^b, c^, Yue Hu ^a, b^,

Kai-Li Lin ^b, c, **^, Yuan-Jin Xu ^a, b, *^

*^a^ Department of* *Oral Surgery, Shanghai Ninth People’s Hospital, Shanghai Jiao Tong University School of Medicine,* *Shanghai, China*

*^b^* *College of Stomatology, Shanghai Jiao Tong University; National Center for Stomatology; National Clinical Research Center for Oral Diseases; Shanghai Key Laboratory of Stomatology; Shanghai Research Institute of Stomatology, Shanghai, China*

*^c^ Department of Oral and Cranio-maxillofacial Surgery, Shanghai Ninth People’s Hospital, Shanghai Jiao Tong University School of Medicine, Shanghai, China*

*∗ Corresponding author. Shanghai Ninth People's Hospital, Shanghai Jiao Tong University School of Medicine, 639 Zhizaoju Road, Shanghai, 200011, China.*

*∗∗ Corresponding author. Shanghai Ninth People's Hospital, Shanghai Jiao Tong University School of Medicine, 639 Zhizaoju Road, Shanghai, 200011, China.*

*E-mail addresses:* ***Corresponding authors:*** *drxuyuanjin@126.com (YJX);* *linkaili@sjtu.edu.cn & lklecnu@aliyun.com (KLL).*

***Other authors:*** *yangshiyuan1996@sjtu.edu.cn (SYY); xiaoyao7958@163.com (YNZ); yuxingge1991@163.com (XGY); 2754036130@qq.com (ZYF); 617167464@qq.com (CCZ); moonhu1992@163.com (YH).*

**Methods S1**

***Materials and regents***

Lyophilized silk fibrin (SF) was provided by Simatech Inc. (Suzhou, China). Gelatin was purchased from Sangon Biotech (Shanghai, China). Genipin was purchased from Aladdin Co., Ltd. (Shanghai, China). Sinopharm Chemical Reagent Co., Ltd. (Shanghai, China) supplied other analytical grade reagents without further purification in this research. Dulbecco’s modified Eagle’s medium (DMEM), fetal bovine serum (FBS), 0.25% trypsin-EDTA, phosphate buffer saline (PBS), and penicillin/streptomycin (P/S) were produced by Gibco (CA, USA). Tetracycline (TE), alizarin red (AL) and calcein (CA) were supplied by Sigma-Aldrich (MO, USA). OriCell Rabbit Bone Marrow Mesenchymal Stem Cell Medium was gained from cyagen (CA, USA). Calcein-AM/ Propidium Iodide (Calcein-AM/PI) double staining kit and Cell Counting Kit-8 (CCK-8) were bought from Dojindo (Tokyo, Japan). BCIP/NBT Alkaline phosphatase color development kit, Bicinchoninic Acid Assay (BCA) protein kit, 2-(4-Amidinophenyl)-6-indolecarbamidine dihydrochloride (DAPI), Actin-tracker green-488, radio-immunoprecipitation (RIPA) lysis buffer and Bovine Serum Albumin (BSA) were manufactured by Beyotime (Shanghai, China). The Alkaline phosphatase (ALP) assay kit manufactured by Nanjing Jiancheng Bioengineering Institute (Nanjing, China) was used in this research. Polyvinylidene fluoride (PVDF) membrane was fabricated by Millipore (MA, USA). Omni-Easy™ One-Step PAGE Gel Fast Preparation Kit and Omni-ECL™ Femto Light Chemiluminescence Kit were manufactured by Shanghai Epizyme Biomedical Technology Co., Ltd (Shanghai, China). TRIzol reagent, PrimerScript™ RT Master Mix kit and TB Green Premix Ex Taq™ kit were made by Takara (Tokyo, Japan).

***Preparation of 3D-printed scaffolds***

**Preparation of CS nanowires:** Xonotlite (Ca_6_(Si_6_O_17_) (OH)_2_, CS) nanowires were synthesized following a previously reported technique^1^. 0.5M Ca (NO_3_)_2_ aqueous solution and 0.5M Na_2_SiO_3_ aqueous solution were firstly prepared and then Na_2_SiO_3_ aqueous solution was dropwise added into Ca (NO_3_)_2_ aqueous solution under strong stirring with Ca/Si=1.0 (mole ratio) to obtain white precipitation, where the reaction system used ammonia water maintains to keep pH above 11. After that, the stainless-steel autoclaves lined with Teflon were used to heat the white precipitation at 200℃ for 24 hours, which then required natural cooling before filtration. Finally, the precipitation was dried for 24 hours at 60℃ to obtain CS nanowires after being thoroughly washed with deionized water and anhydrous ethanol. The phase and the morphology of product were observed by X-Ray Diffraction (XRD; D8A, Bruker, Germany) and transmission electron microscope (TEM; Tecnai G2 F20，FEI, USA), respectively.

**Preparation of inks:** To fabricate the 3D-printed SGC scaffolds, CS was added to the lyophilized SF at weight ratios of 0%, 6.25%, 12.5%, and 25%, resulting in a CS/SF mixture. And then to improve the printability of the materials, we dissolved gelatin powder in deionized water to prepare a 15% (w/v) gelatin solution. After that, the CS/SF mixture was dissolved in gelatin solution and dispersed while continuously magnetically stirring at 37℃ to generate composite inks at an SF/Gelatin weight ratio of 1:1. The rheological properties of the inks was tested by a rheometer (Mars40, Thermo Fisher, USA).

**Fabrication of** **SGC composite scaffolds:** Square-shaped 3D-printed scaffolds measuring 10 × 10 mm and 4 layers in thickness (≈1.65 mm) were fabricated by using a 3D Bioprinter (Bio-Architect^®^ SR, Regenovo, China) through a needle of gage 22 (inner diameter of 0.41 mm) under 0.2-0.25 MPa of extrusion pressure at the printing speed of 4-7 mm/s at 25℃. After printing, the scaffolds were firstly placed in 90% (v/v) ethanol for 10 minutes to crosslink the SF, followed by crosslinking the gelatin by 0.6% (w/v) genipin/ethanol solution at 37 °C for 72 hours^2^. The scaffolds were then freeze-dried at −80°C and the dried scaffolds were stored under ambient conditions until use.

***Characterizations***

**Microscopic morphology****:** The morphology of dried 3D-printed scaffolds was analyzed using a digital camera (SMZ25, Nikon, Japan) and scanning electron microscopy (SEM; JSM-6380LV, JEOL, Japan), while the element distribution and element quantification were analyzed by element mapping and energy EDS.

**Fourier-transform infrared spectroscopy (FTIR)****:** To analyze the structures of the fabricated scaffolds, a FTIR spectrometer (Nicolet iN10, Thermo Scientific, USA) was used to characterize the pre-ground scaffolds at a wavenumber range of 800 - 2000 cm^−1^.

**XRD:** XRD was performed at room temperature from 10° to 60° with a scanning speed of 10°/min.

**Water adsorption, swelling ratio and porosity:** The freeze-dried scaffolds were weighed and recorded as W_0_. And after being fully soaked in PBS for 24 hours at 37℃, filter papers were used to remove the samples’ apparent moisture followed by weighed as W_1_. The swelling ratio and water adsorption rate were calculated according to the following formula:

$$Swelling ratio (\%) =（W_{1}-W_{0})/W_{0}\times100\%$$

$$Water adsorption (\%) =（W_{1}-W_{0})/W_{1}\times100\%$$

The porosity was detected by using a mercury porosimeter (AutoPore Iv 9510, Micromeritics, USA).

**Mechanical properties:** The compressive mechanical property was measured by using a universal testing machine (HY (WE) 100060, Shanghai Heng Wing Precision Instrument co., LTD., China). The linear region of the stress-strain curve was adopted to compute the compressive modulus.

**Degradation behavior characterization****:** To evaluate the in vitro degradation behavior, scaffolds were exposed in SBF at 37 °C in the shaker water bath and the simulated body fluid (SBF) solution was collected in various time points (1, 3, 5, 7, 14 and 21 days). SBF was prepared as described by Kokubo^3,4^. The immersion medium was completely refreshed every 48 hours. The samples’ initial weight was recorded as W_0_. After various soaking time points, the samples were softly washed with deionized water, freeze-dried and the dry weight of each scaffold was recorded as W_d_. The degradation ratio of the scaffolds was calculated based on the following equation:

$$Degradation ratio (\%) =（W_{d}-W_{0})/W_{0}\times100\%$$

The morphologies and elemental compositions of the apatite formed on the lyophilized sample surface immersed for 7 days were examined by SEM, element mapping and energy dispersive spectrometer (EDS) to evaluate the mineralization ability of the fabricated 3D-printed scaffolds.

***Cell isolation and culture***

Four-week-old New Zealand white rabbits were purchased from Shanghai Songjiang District Chedun Experimental Animal Fine Seed Farm Co., Ltd. Animal procedures were approved by the Animal Care and Use Ethics Committee of Shanghai Ninth People's Hospital, Shanghai Jiao Tong University. As previously described, the bone marrow stromal cells (BMSCs) were collected by rinsing the diaphysis of femurs and tibias with PBS which contained 10% FBS and 1% (v/v) P/S. After that, the BMSCs were passaged and maintained in an atmosphere with 5% CO_2_ at 37°C. The culture medium was renewed every 3 days and the adherent cells were passaged until 80%–90% confluence^5^. Cells in the 3-5 passage were harvested for subsequent experiments.

In this study, the RAW 264.7 cell line (Cell Bank of Chinese Academy of Sciences, Shanghai, China) was used to explore the macrophage’s immune response in vitro. RAW 264.7 cells were cultured in DMEM with 10% FBS and 1% P/S. The cells were executed passage cultures after measuring up to about 90% confluence.

***Cell viability and adhesion***

The viability of BMSCs was analyzed after 1, 4, and 7 days, and the viability of RAW264.7 was analyzed after 1 and 3 days using CCK-8 assays. Optical density (OD) was measured at 450 nm using a microplate reader (Spectra-Max M5, Molecular Devices, USA). Calcein-AM/ PI staining was used to further obverse the survival of the BMSCs (performed on 4, 7 days) and RAW264.7 (performed on 1, 3 days) .

To visualize the cell adhesion on the scaffolds, at the same time node like calcein-AM/ PI staining, samples co-cultured with BMSCs were firstly fixed with 4% paraformaldehyde (PFA). After that, the samples were stained by using actin-tracker green-488 and DAPI. CLSM (Leica, Germany) was employed to picture fluorescent images.

***Assessment of*** ***BMSCs osteo******-******/angiogenic differentiation and macrophage polarization***

**Osteo****-/angiogenic differentiation of BMSCs on 3D-printed scaffolds****:** After the BMSCs were cultured with scaffolds for 4, 7 and 10 days, total RNA was extracted using the TRIzol reagent and Prime Script RT Master Mix according to the manufacturer's instructions. Quantitative real-time polymerase chain reaction (qRT-PCR) was performed with TB Green Premix ExTap to analyze the mRNA expression levels of the target genes related to osteo-/angiogenic differentiation. The primer sequences used in this section were listed in *Supplemental Table S.1* and GAPDH was used as endogenous standards. The 2^-ΔΔCT^ method was utilized to calculate relative expression of the target genes. After co-culture for 7 days, immunofluorescence staining was performed to reveal the expression of osteogenic protein. Briefly, cellular samples were fixed and then incubated with OPN antibody (1B20, NOVUS, USA) at 4 °C overnight. Next, the cells were processed with Alexa Fluor 594-conjugated Donkey Anti-Mouse IgG H&L (ab150108, abcam, UK) for 1 hour at room temperature. Actin-tracker green-488 and DAPI were used for stamping cytoskeleton and nuclei. The cells were photographed with fluorescence microscope (DS-Ri2, Nikon, Japan).

**Polarization of macrophage on 3D-printed scaffolds:** After 1 and 3 days of co-culture, qRT-PCR was used to assess the expression of the pro-inflammatory genes and anti-inflammatory genes described in *Supplemental Table S.2* and β-actin was selected as the reference gene.

***Influence of 3D-printed scaffolds co-cultured macrophages on the osteo-/angiogenic differentiation of BMSCs***

**Condition mediums (CMs) collection:** To obtain the supernatant from macrophages, RAW264.7 (1× 10^5^ cells/well in 6-well plates) was seeded on scaffolds. The scaffolds were immersed in DMEM without cell seeding as controls for material dissolution. The culture media collected on day 3 was filtered by 0.22 μm filter to acquire CM and stored in -80℃ for future use. Before use, CM was mixed with DMEM culture medium at a ratio of 1:1 (v/v) and divided as following groups: (1) Control, (2) SG; (3) SGC_M_; (4) MΦ; (4) MSG; (6) MSGC_M_.

**Proliferation of BMSCs with the CMs:** The BMSCs seeded in 96-well plates were incubated with the corresponding CM for 1, 4 and 7 days followed by the CCK-8 assay as described above.

**Cell migration:** Transwell assay and scratch test were performed to determine the migratory ability of BMSCs cultured in CMs. Transwell assay was performed as follows: the BMSCs suspension (2×10^4^cells/well) in serum-free DMEM was put into the upper transwell compartment and then 650 μL CM (n=3) was placed in the lower chamber. The migrating cells were stained with crystal violet after cultured for 24 hours.

Scratch test was performed by seeding BMSCs 2×10^5^ cells /well in each twelve-well plate. And until confluent rate was up to 100%, cell‐free strips were made with a 10 μL pipet tip and recorded as 0 hours. After that, the cells were incubated for another 24 hours and the migrated cells in cell‐free strips were pictured by the microscope. The number of migrating cells stained with crystal violet and the change in scratch area were all quantitated by ImageJ software (NIH Image, USA).

**ALP and** **ARS staining:** ALP staining and ALP activity were performed according to the description in the manual after the BMSCs cultured for 7 days with CM. BMSCs were mineralized for 14 and 21 days, then stained with ARS solution (1%, PH 4.2) for 30 minutes. The precipitated ARS was imaged was dissolved and dissolved solutions with 10% cetylpyridinium chloride of the samples were quantitatively measured at 562 nm.

**Osteo-/angiogenic gene expression of BMSCs:** The mRNA expression of osteogenesis related genes (BMP-2 and OPN) and angiogenesis related gene (VEGF) was detected by qRT-PCR after cultured for 7 days. The technology and primer sequences were carried out as described above

**Osteo-/angiogenic protein expression of BMSCs:** The protein expression of OPN and VEGF was determined by western blot according to the instructions^6^. Briefly, RIPA lysis was applied to collect the protein of BMSCs after cultured for 7 days. After quantified by BCA protein assay kit, protein samples were electrophoresed in PAGE gels and transferred to PVDF membrane. Then, the PVDF membrane was firstly blocked with 5% BSA, then incubated with the OPN, VEGF (MA5-13182, Thermo Fisher Scientific, USA) and GAPDH (60004-1-Ig, proteintech, China) antibody at 4 °C overnight and finally immersed in secondary antibody at room temperature for 1 hour. A chemiluminescence imager (Amersham600, cyvita, USA) was employed to assess the immunoreactive bands using the chemiluminescence kit. The protein bands were semi-quantified by ImageJ software.

**Osteo-/angiogenic immunofluorescence staining:** After being cultured for 14 days, the cellular samples were fixed and incubated overnight at 4 °C with OPN and VEGF antibodies. Immunofluorescence staining was performed following the same steps as previously described.

***RNA Sequencing***

RAW264.7 cells cultured with different scaffolds were collected in triplicate after 3 days for RNA sequencing. The total RNA was extracted using TRIzol reagent and and sent to the BerryGenomics Co. (Beijing, China). The Illumina Novaseq 6000 sequence platform performed the cDNA libraries construction, sequencing and bioinformatic analysis. The DGEs were determined on the basis of the |log2 (Fold Change) | ≥ 1 & q value ≤ 0.05. RT-qPCR was used to confirm the immune mechanism of SGC_M_ hydrogel.

***Animal study***

**Study design:** Eighteen 7-month-old male New Zealand White rabbits were operated to create calvarial defects. Overall process was approved by the Independent Ethics Committee of Shanghai Ninth People's Hospital affiliated with Shanghai JiaoTong University, School of Medicine (SH9H-2019-A294-1).

**Implantation surgery****:** After anesthesia, healthy white rabbits were operated with a ring drill on a low-speed dental handpiece, and washed with plenty of saline solution, thus creating a 7 mm complete round defects on the left and right sides of the parietal bone area. Next, 3D printed scaffolds were placed into the defects area and the untreated skull defects were set as blank group. The rabbits were divided into three groups as follows: (1) blank (defects filled with nothing); (2) SG (defects filled with SG scaffolds); (3) SGC_M_ (defects filled with SGC_M_ scaffolds). At 4, 8, and 12 weeks after operation, TE (25 mg/kg), AL (30 mg/kg) and CA (20 mg/kg) were respectively injected into the abdominal cavity of rabbits. All the rabbits were euthanized at 16 weeks after surgery.

**Micro-CT analysis:** After euthanized with CO_2_ inhalation, calvarial samples were fixed in 4% PFA and subsequently scanned by micro-CT (Quantum GX, PerkinElmer, USA). The bone regeneration pattern at region of interest (ROI) was computed as BV/TV.

**Sequential fluorescent labeling:** After micro-CT analysis, some samples were sectioned into 10 μm slices. Three staining results for TE, AL and CA were captured by confocal laser scanning microscopy (CLSM) and analyzed by ImageJ software.

**VG staining and** **masson's trichrome staining:** VG staining and masson's trichrome staining were performed to assess the volume of newly formed bone and the values among samples were calculated by using ImageJ software.

**H&E staining and immunohistochemistry analysis:** After decalcification in 10% ethylenediaminetetraacetic acid (EDTA) for 4 weeks, calvarial samples were embed in paraffin followed by sliced into 5 μm-thick sections. H & E staining was performed following the manufacturer's instructions. Images of the stained slides were captured using NanoZoomer Digital Pathology (Hamamatsu, Japan). Another portion of slices was used for immunohistochemistry analysis using IL-1β (ab156791, abcam, UK), TGF-β (ab190503, abcam, UK), OPN and VEGF, followed by capture under a light microscope. ImageJ software was used to count the integrated optical density (IOD) of positive cells in 200× images (n = 5).

***Statistical analysis***

The quantitative data was shown as means ± standard deviation (SD). One-way analysis of variance (ANOVA) was utilized to perform statistical analysis and the level of the significant differences between groups was set as **P < 0.05, **P < 0.01 or ***P < 0.001.* GraphPad Prism 7.0 was used for statistical analysis.

**Results**

**Table S1** The sequences of BMSCs genes (F: forward primer R: reverse primer).

| Gene | Full name | Primer | Primer nucleotide sequence (5’-3’) |
| --- | --- | --- | --- |
| GAPDH | Glyceraldehyde-3-phosphate dehydrogenase | F | GGGTGGTGGACCTCATGGT |
|  |  | R | CGGTGGTTTGAGGGCTCTTA |
| BMP-2 | Bone morphogenetic protein-2 | F | CGTGAGGATTAGCAGGTCTTTG |
|  |  | R | CCCTTGACGCTTTTCTCTTCTG |
| COL-1 | Collagen I | F | GCCACTCTGAAGTCTCTGAACAAC |
|  |  | R | TAGTAACCACTGCTCCACTCTGG |
| OCN | Osteocalcin | F | TCTACCAGTTGCAGCCTGAC |
|  |  | R | GTTCCCTTCCTCCTTGATTT |
| OPN | Osteopontin | F | CACTGAAGTCGTTCCCACAGTAG |
|  |  | R | GTATCATCCAAGTCCTCGCTGTC |
| Osteonectin | Osteonectin | F | GCAGCAATGACAACAAGAC |
|  |  | R | GCTTCTCATTCTCATGGATC |
| RUNX-2 | Runt-related transcription factor 2 | F | TCAGGCATGTCCCTCGGTAT |
|  |  | R | TGGCAGGTAGGTATGGTAGTGG |
| bFGF | Basic fibroblast growth factor | F | CAAGCGGCTGTACTGCAAAAA |
|  |  | R | AGCAAGGTAACGGTTTGCAC |
| VEGF | Vascular endothelial growth factor | F | CGAGACCTTGGTGGACATC |
|  |  | R | CTGCATGGTGACGTTGAAC |

**Table.S.2** The sequences of macrophage genes (F: forward primer R: reverse primer).

| Gene | Full name | Primer | Primer nucleotide sequence (5’-3’) |
| --- | --- | --- | --- |
| IL-1β | Interleukin-1β | F | GAAATGCCACCTTTTGACAGTG |
|  |  | R | TGGATGCTCTCATCAGGACAG |
| IL-6 | Interleukin-6 | F | TAGTCCTTCCTACCCCAATTTCC |
|  |  | R | TTGGTCCTTAGCCACTCCTTC |
| IL-10 | Interleukin-10 | F | GCTCTTACTGACTGGCATGAG |
|  |  | R | CGCAGCTCTAGGAGCATGTG |
| TGF-β | Transforming growth factor-β | F | CTCCCGTGGCTTCTAGTGC |
|  |  | R | GCCTTAGTTTGGACAGGATCTG |
| β-actin | β-actin | F | GGCTGTATTCCCCTCCATCG |
|  |  | R | CCAGTTGGTAACAATGCCATGT |

**
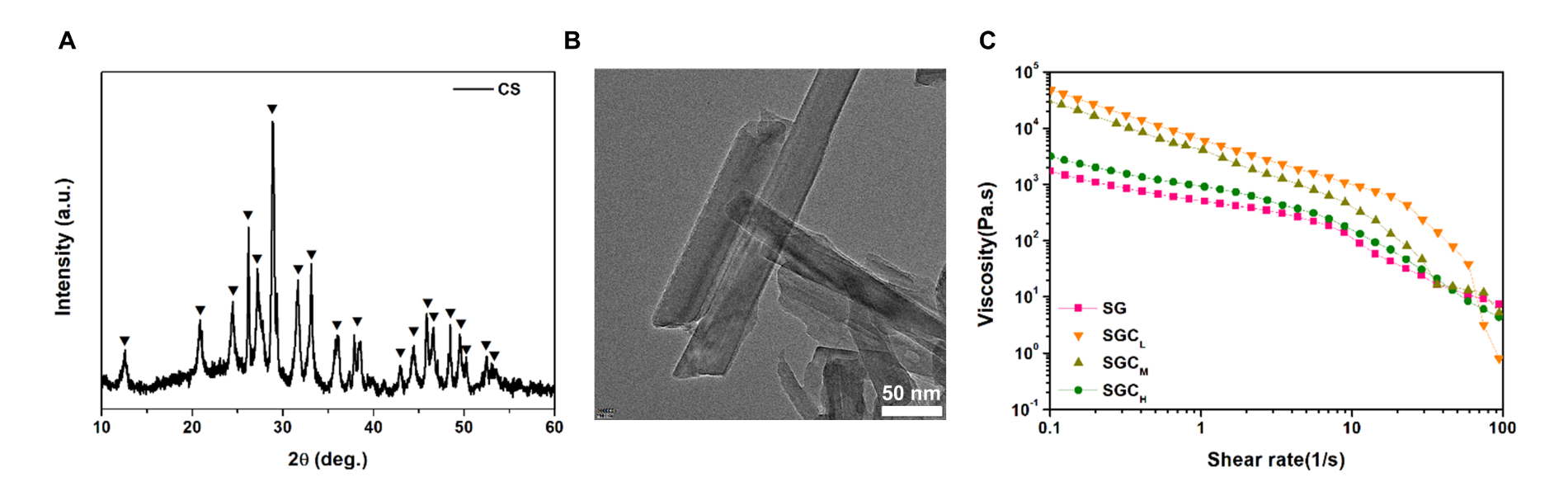
**

*Fig. S1* ***A-B*** *XRD and TEM of CS.* ***C*** *The Viscosity-shear rate curve of SG, SGC_L_, SGC_M_ and SGC_H_.*

*
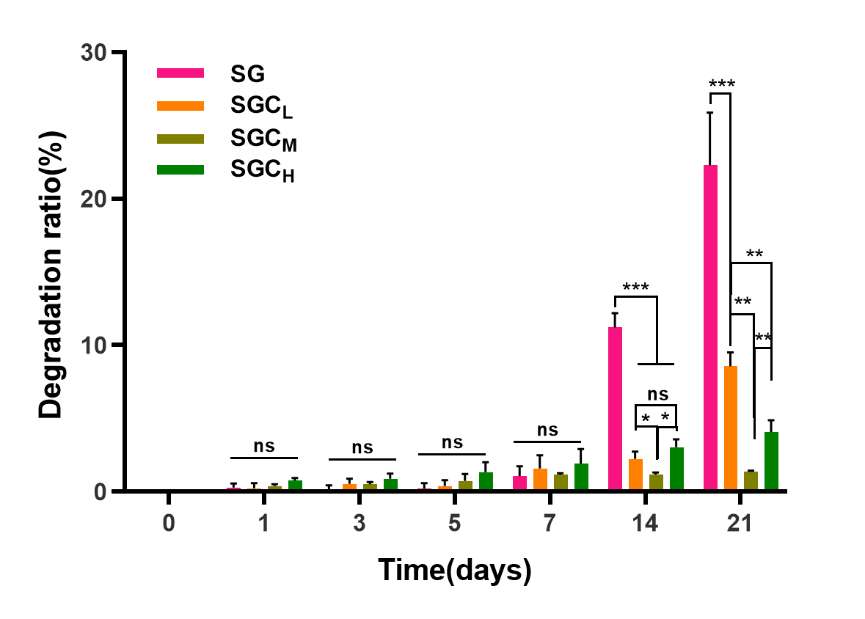
*

*Fig. S2 The degradation ratio of SG, SGC_L_, SGC_M_ and SGC_H_ samples in histogram.* *^*^P < 0.05, ^**^P < 0.01 or ^***^P < 0.001.*

*
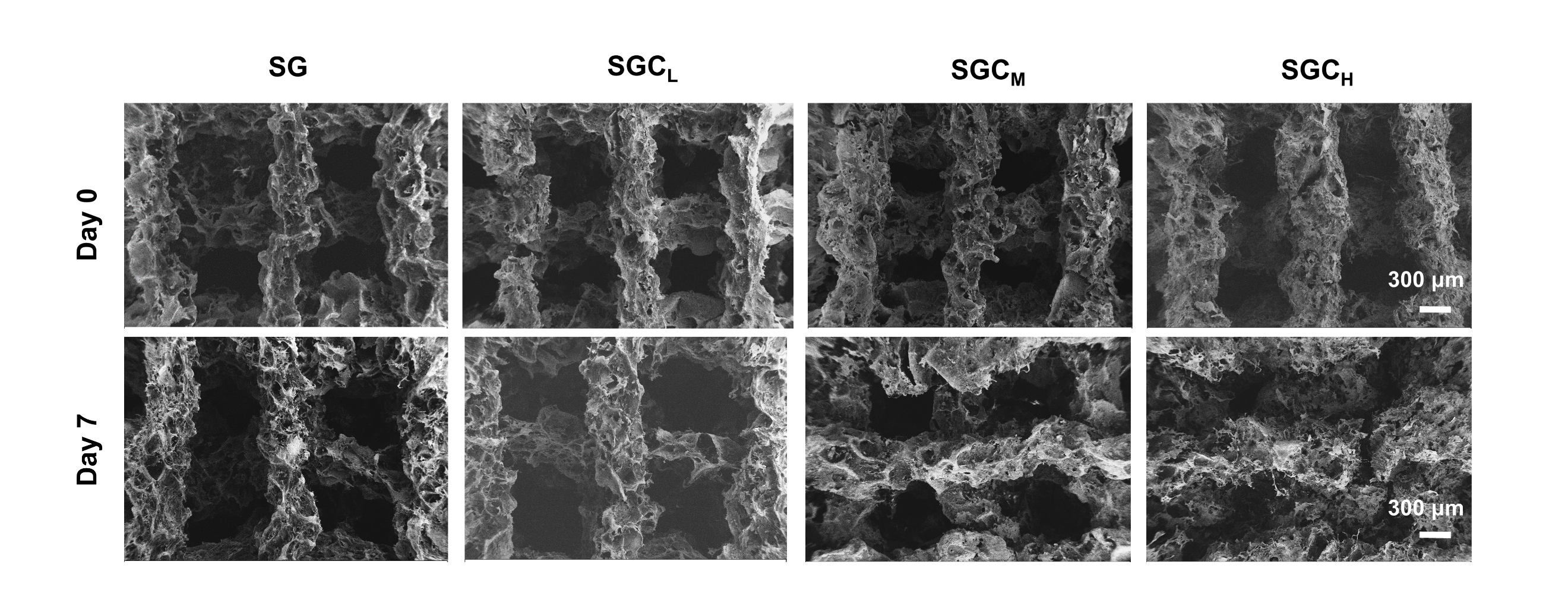
*

*Fig. S3 SEM photographs in low magnification lens of SG, SGC_L_, SGC_M_ and SGC_H_ sampled after degradation for 7 days.*

*
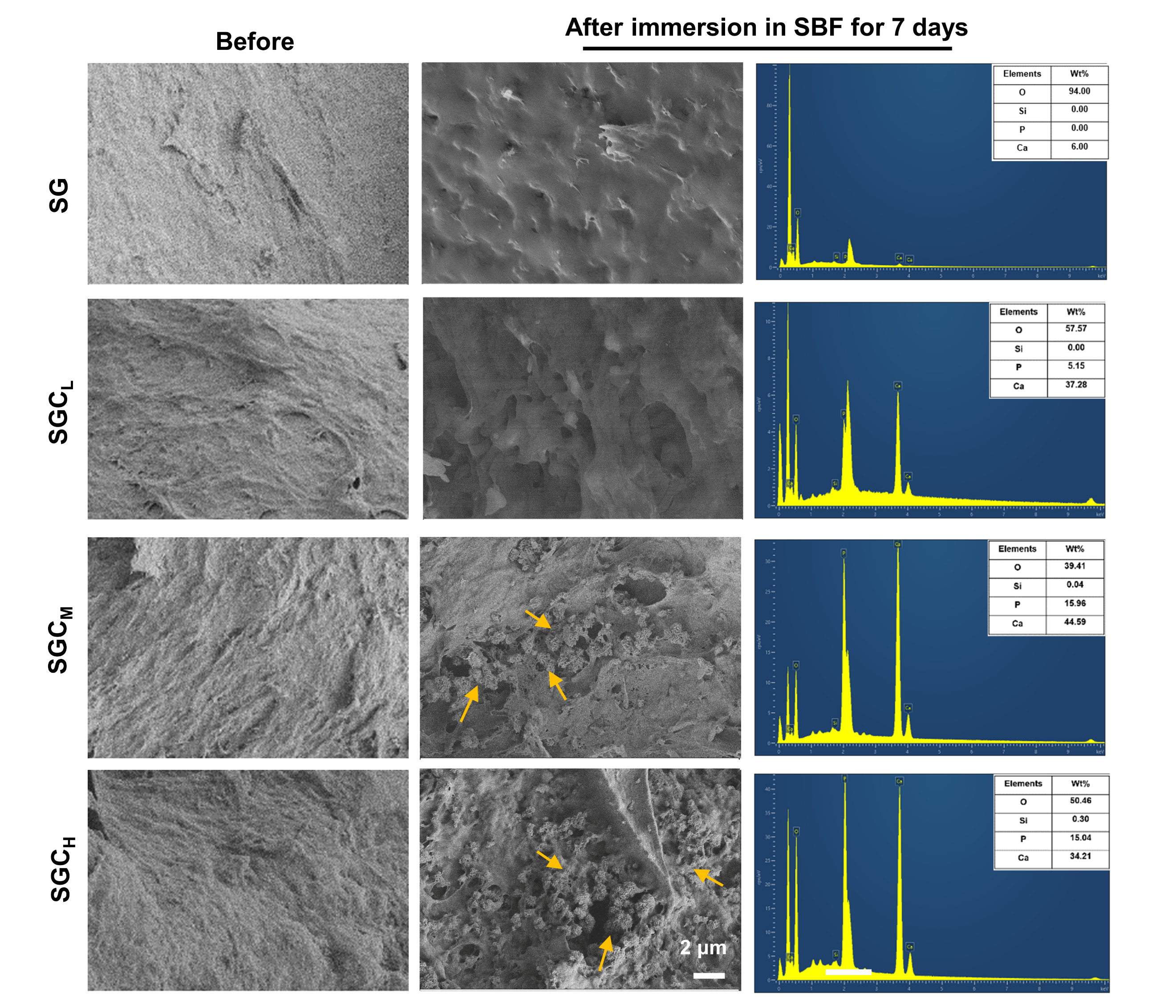
*

*Fig. S4* *SEM photographs in high magnification lens and EDS analysis of composite specimens with different CS contents immersed in SBF (yellow arrow marks the apatite spherulites).*

*
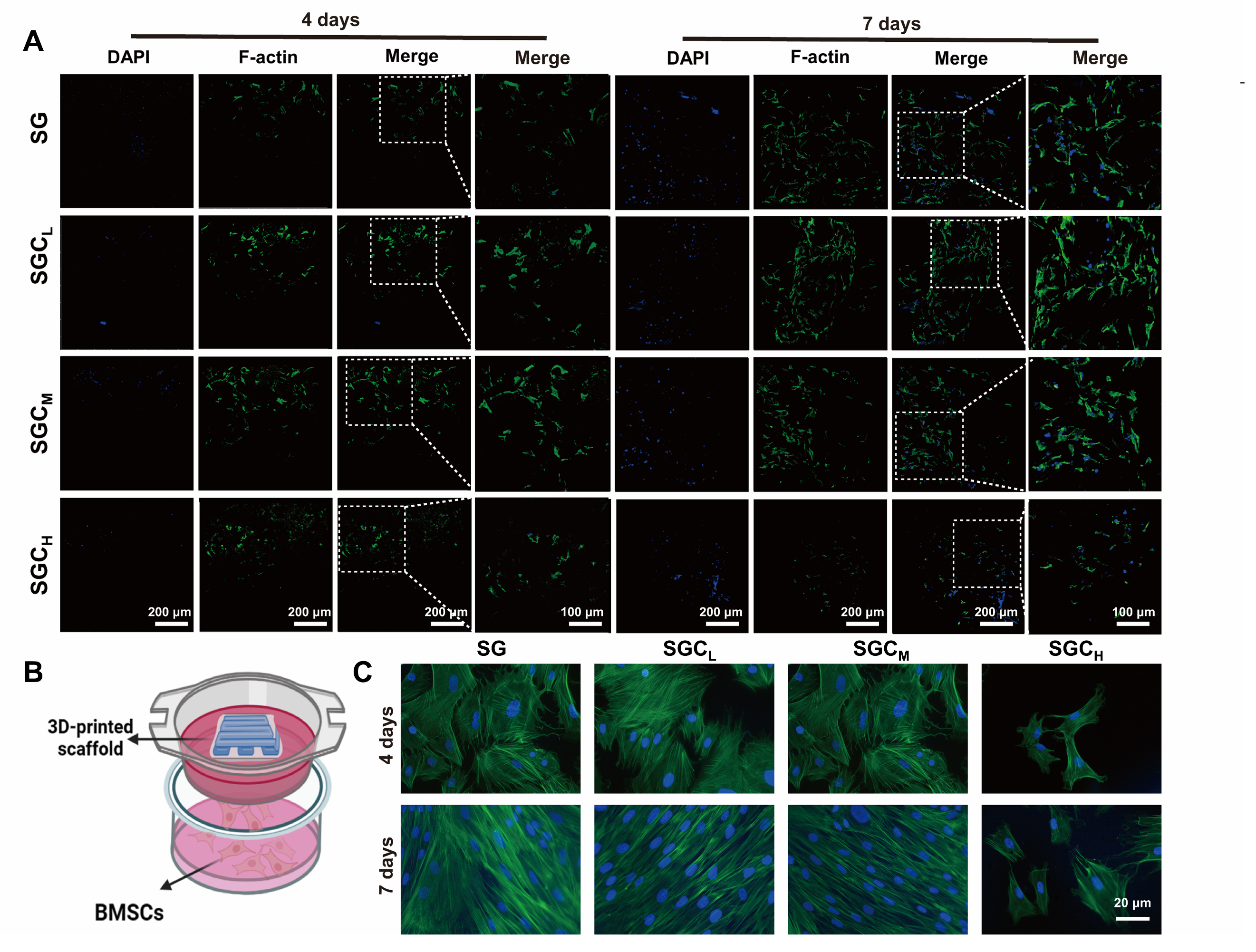
*

*
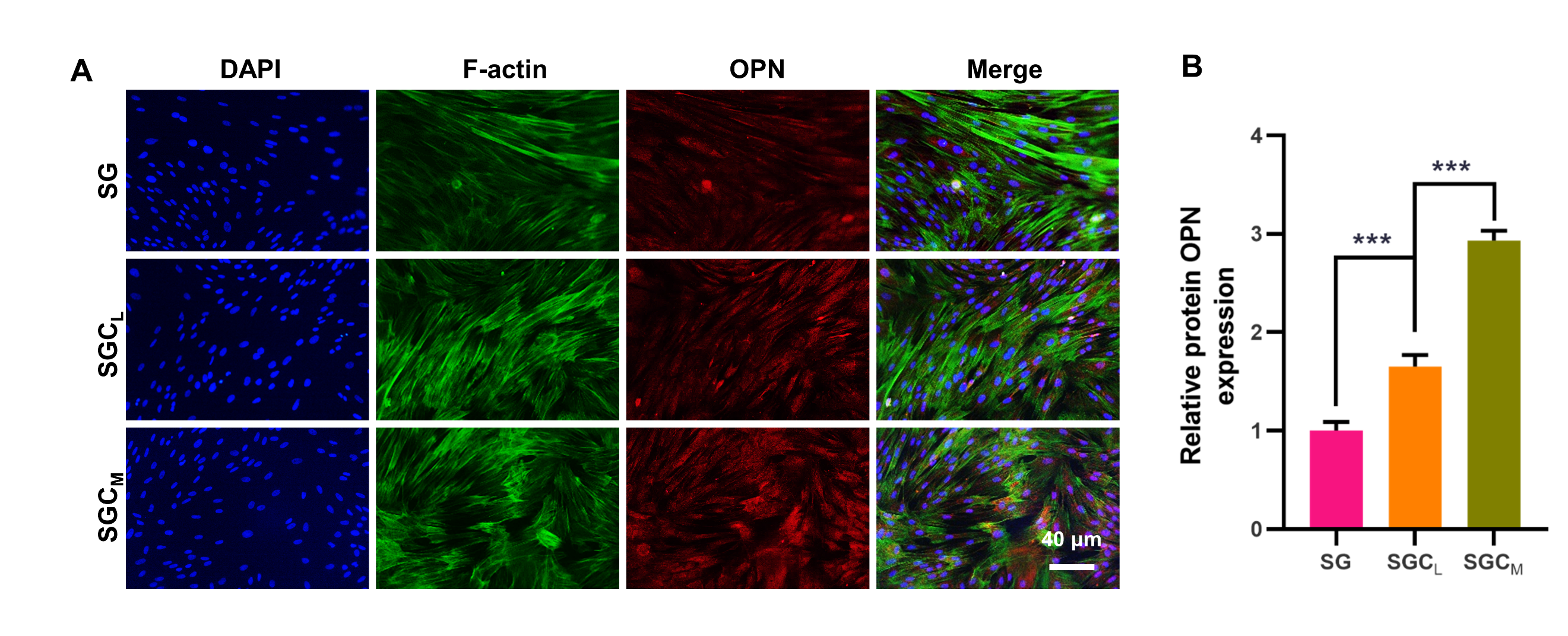
Fig. S5* ***A*** *The F-actin/DAPI staining of BMSCs* *"co-culture on" SG, SGC_L_, SGC_M_ and SGC_H_ scaffolds.* ***B*** *Schematic illustration of the co-culture models- "co-culture with" hydrogels.* ***C*** *The F-actin/DAPI staining of BMSCs "co-culture with" SG, SGC_L_, SGC_M_ and SGC_H_ scaffolds.*

*Fig. S6* ***A-B*** *The immunofluorescence staining and its quantitative assay of the OPN protein in BMSCs after cultured for 7 days. ^*^P < 0.05, ^**^P < 0.01 or ^***^P < 0.001.*

**Reference**

1. Lin K, Liu X, Chang J, Zhu Y. Facile synthesis of hydroxyapatite nanoparticles, nanowires and hollow nano-structured microspheres using similar structured hard-precursors. *Nanoscale*. 2011;3:3052-5.

2. Wei L, Wu S, Kuss M, Jiang X, Sun R, Reid P, et al. 3D printing of silk fibroin-based hybrid scaffold treated with platelet rich plasma for bone tissue engineering. *Bioact Mater*. 2019;4:256-260.

3. Kokubo T, Kushitani H, Sakka S, Kitsugi T, Yamamuro T. Solutions able to reproduce in vivo surface-structure changes in bioactive glass-ceramic A-W. *J Biomed Mater Res*. 1990;24:721-34.

4. Kokubo T, Takadama H. How useful is SBF in predicting in vivo bone bioactivity? *Biomaterials*. 2006;27:2907-15.

5. Abdallah BM, Kassem M. The use of mesenchymal (skeletal) stem cells for treatment of degenerative diseases: Current status and future perspectives. *Journal of cellular physiology*. 2009;218:9-12.

6. Wu M, Chen F, Liu H, Wu P, Yang Z, Zhang Z, et al. Bioinspired sandwich-like hybrid surface functionalized scaffold capable of regulating osteogenesis, angiogenesis, and osteoclastogenesis for robust bone regeneration. *Mater Today Bio*. 2022;17:100458.
